# The changing landscape of outpatient antibiotic prescriptions among advanced practice clinicians in the United States, 2011 and 2022

**DOI:** 10.1017/ash.2026.10346

**Published:** 2026-06-01

**Authors:** Mohsin Ali, Guillermo V. Sanchez, Katryna A. Gouin, Emily McDonald, Adam L. Hersh, Sarah Kabbani

**Affiliations:** 1 Centers for Disease Control and Prevention, USA; 2 University of Utah, USA

## Abstract

**Objective:**

Estimate changes in antibiotic prescribing among advanced practice clinicians (APCs)—nurse practitioners (NPs) and physician assistants (PAs)—compared to physicians in 2022 versus 2011 to inform antibiotic stewardship efforts.

**Methods:**

Retrospective descriptive analysis of antibiotic prescription rates for 2011 and 2022 using county-level prescription dispensing data by provider type from IQVIA Xponent® (numerator) and population census estimates (denominator). Prescribing rates among physicians and APCs (NPs and PAs) nationally, by state, and by rurality of county are reported.

**Result:**

Overall outpatient antibiotic prescribing rates in 2022 declined by 19.2% compared to 2011, from 877 to 709 prescriptions per 1000 population. The physician rate declined by 45% (628 to 345 per 1000 population), whereas the rate for APCs rose by 104% (124 to 253 per 1000 population), particularly for NPs (148% increase from 65 to 161 per 1000 population). The increase in NP prescribing rates was distinctly higher in seven contiguous Southeastern states (163 to 393 per 1000 population from 2011 to 2022, respectively), where rates were higher within rural counties (range, 385 to 651 per 1000 population by state in 2022).

**Conclusion:**

APCs accounted for 1 in 3 outpatient antibiotic prescriptions in 2022, more than doubling their rate per capita over the past decade. This increase was especially prominent for NPs, particularly within the Southeast region, likely reflecting their growing role as rural primary care clinicians. Integration of APCs for outpatient antibiotic stewardship efforts is essential.

## Background

Antibiotic stewardship is the effort to measure and improve the use of antibiotics by clinicians and patients.^1^ In the United States, most antibiotics are prescribed in outpatient settings, where advanced practice clinicians (APCs)—nurse practitioners (NPs) and physician assistants (PAs)—are a critical and growing part of the healthcare workforce. Over the past decade, the number of APCs has more than doubled, reaching over 500,000 in 2022, with more than 70% (355,000) of them being NPs.^2,3^ In 2020, seven in ten NPs were certified to practice in primary care, accounting for up to one in four providers in rural areas.^4,5^ In contrast, only 23% of PAs practiced in primary care settings in 2022, and over 90% of PAs overall practiced in urban settings in 2022.^3^

Over the past decade, APCs accounted for a growing proportion of antibiotic prescriptions,^6^ especially in rural areas^7–9^. Research findings have been mixed when comparing the appropriateness of prescribing between APCs and physicians.^7,10–19^ An improved understanding of antibiotic prescribing among APCs can inform efforts to tailor antibiotic stewardship messaging and best practices to this clinician audience. We analyzed rates of outpatient antibiotic prescriptions among physicians, NPs, and PAs across the U.S. in 2011 and 2022, by state and by the rurality of the county.

## Methods

We conducted an ecological study using annual county-level antibiotic prescriptions in 2011 and 2022, extracted from the IQVIA Xponent® database. IQVIA uses a proprietary projection method to estimate 100% of prescriptions dispensed at U.S. retail pharmacies, based on a sample of 74% of outpatient prescriptions for 2011 and 93% for 2022.^20^ Unlike CDC’s annual Outpatient Antibiotic Prescribing Report that is based on *per-capita* rates derived from IQVIA Xponent® dispensing data, this analysis was limited to 2011 and 2022 given availability of the provider-type denominator used to calculate rates *per average provider* for a given provider type (see next paragraph). Counties were classified as urban or rural using the most recent National Center for Health Statistics (NCHS) classification scheme, with “non-metropolitan” counties considered rural.^21^

We calculated three types of rates by provider type (physicians, NPs, and PAs). First, to determine population-based rates of antibiotic prescribing, we calculated *per capita* prescribing rates using U.S. census denominators for 2011 and 2022. Second, to account for variability in number of prescribers, we calculated prescribing rates *per average provider* for 2022, representing the average (mean) number of antibiotic prescriptions per provider for that provider type.^8,20^ Provider specialties as provided by IQVIA were based on the American Medical Association self-designated practice specialties; NPs and PAs were categorized as such, regardless of practice specialty. Counts of providers by specialty were aggregated at the ZIP code level and matched to the corresponding county using the U.S. Department of Housing and Urban Development–United States Postal Service ZIP Code Crosswalk Files.^22^ Third, to account for *both* the number of providers and population, we calculated a combined rate *per average provider per capita* for 2022, representing the average (mean) number of prescriptions per provider for that provider type, for every 100,000 population.

We examined spatiotemporal trends in antibiotic prescribing rates by state between provider types. For temporal trends, we used Pearson’s correlations to compare per-capita rates by provider type with the state’s overall rate in 2011 and 2022. For spatial (geographic) trends, we compared all three types of rates between rural and urban counties by state in 2022, further depicting rural–urban rate differences between physicians and NPs in choropleth maps (given that the temporal analysis demonstrated that these two provider types accounted for most of the per-capita antibiotic prescribing rate by state). Geographic analyses were limited to the 47 states with rural counties (ie, excluding the District of Columbia (DC), Delaware, New Jersey, and Rhode Island). Finally, we repeated these rural–urban comparisons of prescribing rates for four antibiotics: amoxicillin, azithromycin, cefdinir, and ciprofloxacin. These agents were selected as they were among the top 10 most commonly prescribed antibiotics in both years. Specifically, azithromycin, ciprofloxacin, and cefdinir are broader spectrum, second-line agents for common outpatient infectious syndromes; thus, their use may indicate inappropriate prescribing.^20,23^

Statistical analyses were conducted using SAS 9.4, and data visualizations were created using Stata 17. All statistical tests were two-sided; *P* less than .05 was considered statistically significant. This activity was reviewed by CDC and was conducted consistent with applicable federal law and CDC policy.^1^

## Results

**Figure 1.**
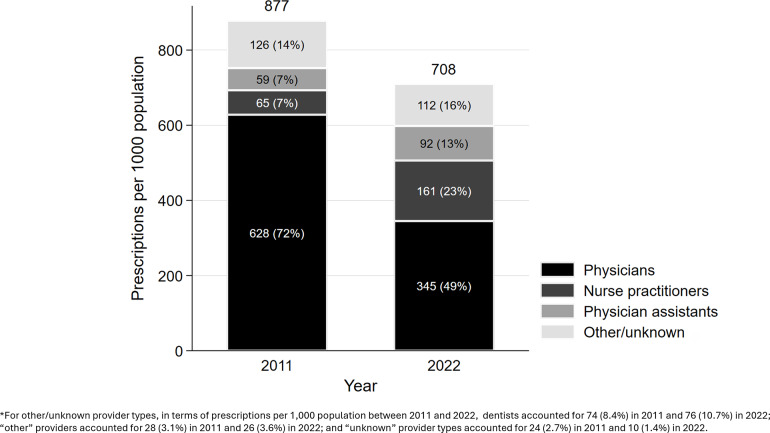
Outpatient antibiotic prescription rates per capita by provider type, 2011 and 2022. Percentage in parantheses refers to provider-type-specific proportion. Data source: IQVIA Xponent®.

Overall, outpatient antibiotic prescribing rates per capita in 2022 declined by 19.2% compared to 2011 (877 to 709 prescriptions per 1,000 population). Physicians accounted for 54% of all outpatient antibiotic prescribers in 2011, compared to 19% for APCs (Figure 1 and Table S1). In 2022, physicians accounted for 40% of outpatient antibiotic prescribers, compared to 25% for APCs. Physicians’ antibiotic prescribing rate declined by 45% during that time frame (628 to 345 prescriptions per 1,000 population), whereas the APC prescribing rate rose by 104% (124 to 253 prescriptions per 1,000 population). In particular, the rate among NPs increased by 148% (65 to 161 prescriptions per 1,000 population). The magnitude of the decline in physician prescribing rates and increases in NP and PA prescribing rates varied by patient age group and antibiotic categories (Table S2).

**Figure 2.**
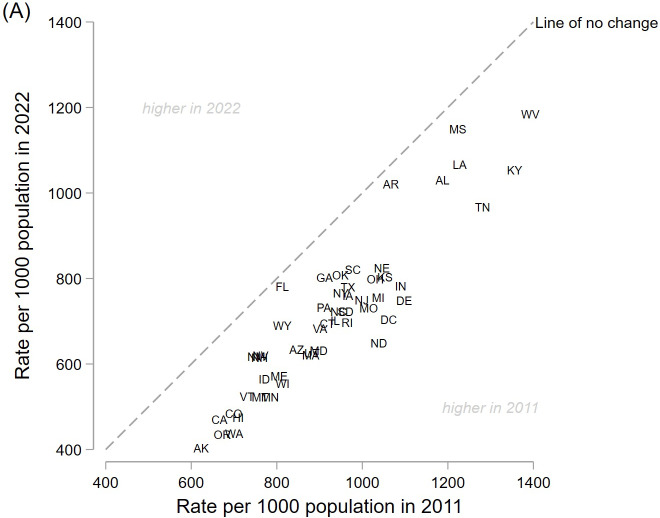
Outpatient antibiotic prescription rates per capita rate by state, 2011 versus 2022, for all provider types. Data source: IQVIA Xponent®.

Outpatient antibiotic prescribing rates per capita declined in all 50 states and Washington, DC, between 2011 and 2022, to varying degrees. In particular, seven contiguous states within the Southeast region—Alabama, Arkansas, Kentucky, Louisiana, Mississippi, Tennessee, and West Virginia—had relatively higher prescribing rates in both years (range, 1,031–1,354 per 1,000 population in 2011 and 967–1,184 per 1,000 population in 2022; Figure 2 and Table S1). By provider type in those seven higher prescribing states, the physician rate more than halved when comparing 2011 and 2022 (cumulatively declining by 53%, from 837 to 445 prescriptions per 1,000 population), while the NP rate more than doubled (cumulatively rising by 133%, from 163 to 393 prescriptions per 1,000 population), a distinctive trend for those states (Figure S1). Per-capita rates for PAs changed minimally by state between 2011 and 2022.

**Figure 3.**
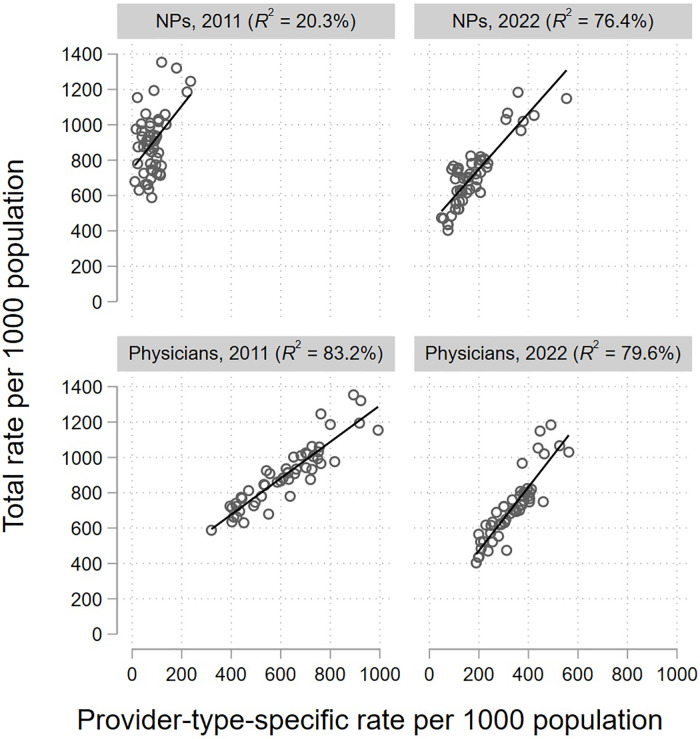
Outpatient antibiotic prescriptions per capita by physicians and nurse practitioners as a predictor of the overall rate by state — 2011 and 2022. Data source: IQVIA Xponent®.

Physicians’ antibiotic prescription rate was a strong predictor of the total rate by state in both years (*R* ^2^ = 0.83, *P* < .0001 in 2011 and *R* ^*2*^ = 0.80, *P* < .0001 in 2022; Figure 3). Conversely, the NP prescription rate explained only 20% of variability between states in 2011 (*R* ^2^ = 0.20, *P* = .0009) but was comparable to that of physicians in 2022 (*R* ^2^ = 0.76, *P* < .0001). The PA prescription rate was a comparatively poor predictor of the total rate by state in both years (*R* ^2^ = 0.03, *P* = .24 in 2011; *R* ^2^ < 0.01, *P* = .90 in 2022).

**Figure 4.**
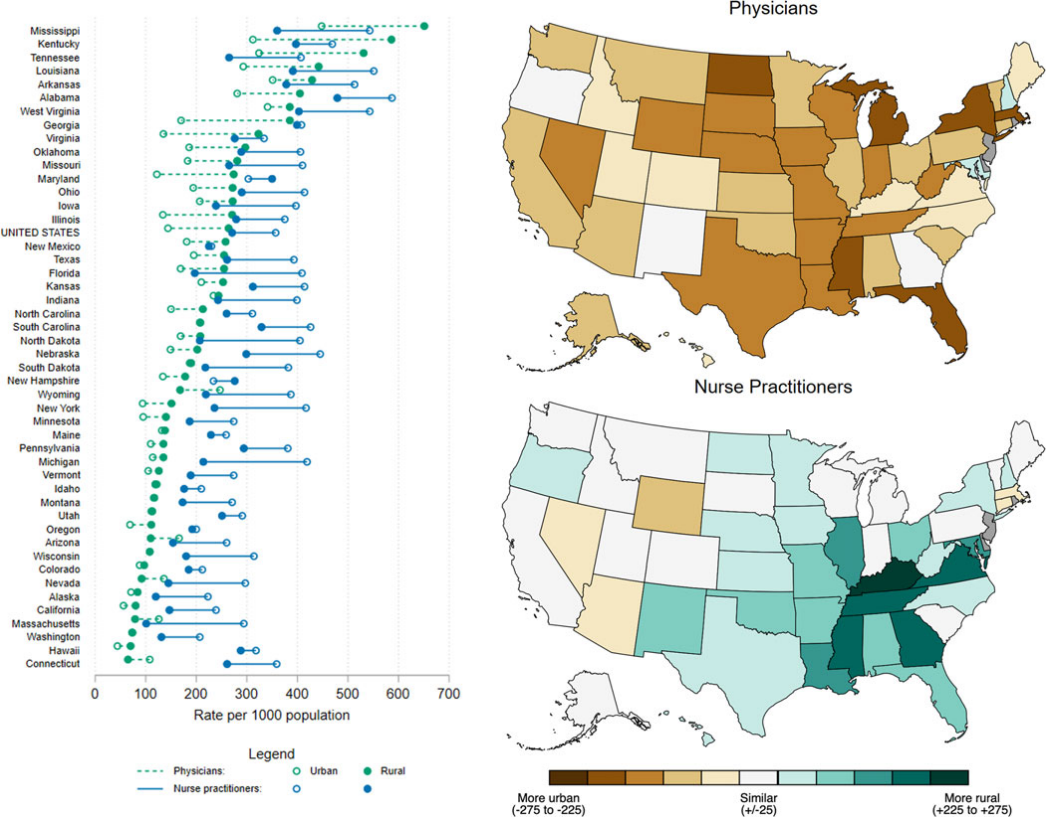
Rural and urban prescription rates per capita for physicians and nurse practitioners, by state. Dumbbell plot sorted by rural nurse-practitioner rate, with the highest at the top. Choropleth maps depict rural–urban rate difference. Data source: IQVIA Xponent®.

Overall prescribing rates per capita were similar for rural and urban counties in 2022 (707 and 709 prescriptions per 1,000 population, respectively). Nationally, 7.2% (60,327) of physicians practiced in rural counties, compared to 12% (42,876) of NPs and 9.4% (14,495) of PAs. Physicians’ prescribing rate per capita was 32% higher in urban counties (357 vs 271 prescriptions per 1,000 population), whereas NPs’ prescribing rate was 83% higher in rural counties (264 vs 144 prescriptions per 1,000 population); rural and urban prescribing rates were comparable for PAs (91 vs 92 prescriptions per 1,000 population, respectively; Table S2). Physicians had higher prescribing rates per capita nationwide, without a discernible geographic trend by state (Figure 4). For NPs, however, rates per capita were distinctly higher in the rural counties of the seven highest-prescribing states in the Southeast region—both in terms of the magnitude of the rate (range, 385 in West Virginia to 651 in Mississippi per 1,000 population) as well as the rate difference (rural–urban rate difference per 1,000 population, 44 in West Virginia to 274 in Kentucky; Figure 4 and Table S3).

**Figure 5.**
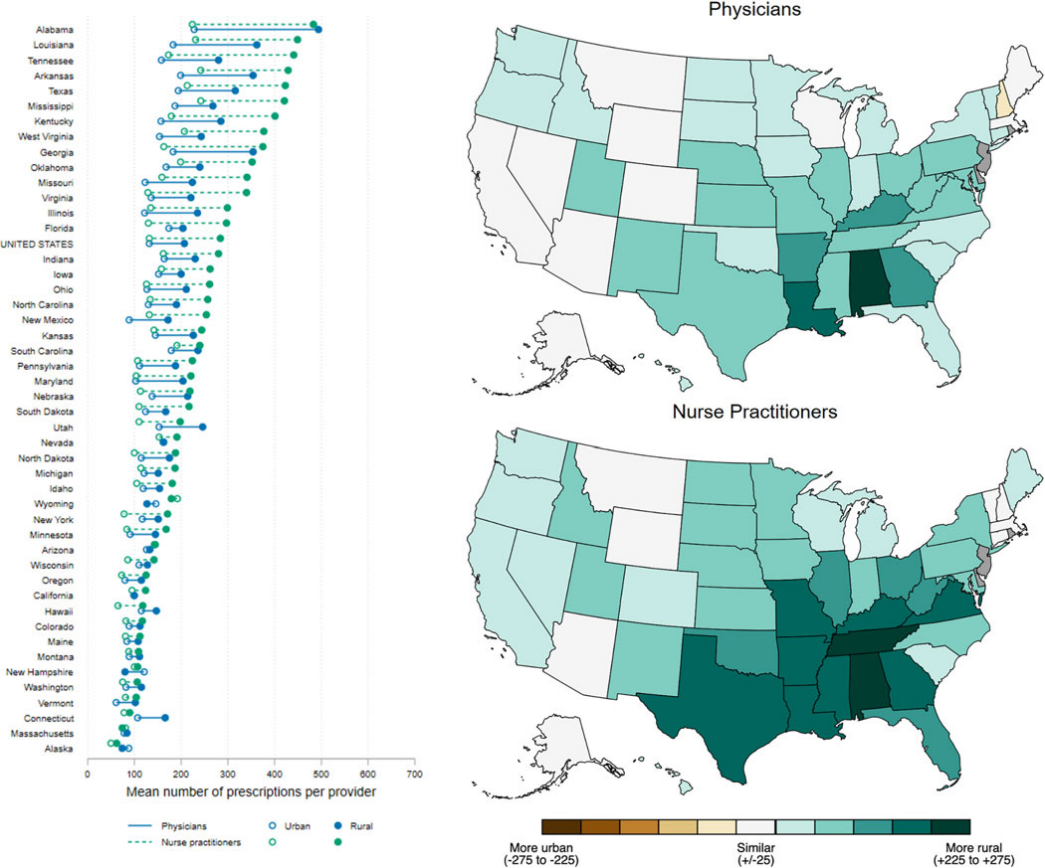
Rural and urban prescription rates per provider for physicians and nurse practitioners, by state. Dumbbell plot is sorted by rural nurse-practitioner rate, with the highest at the top. Choropleth maps depict rural–urban rate difference. Data source: IQVIA Xponent®.

In the same seven higher-prescribing states, the average physician and NP prescribed more antibiotics in rural compared to urban counties in 2022. Nationally, the average rural physician prescribed 75 (57%) more antibiotic courses compared to the average urban physician (207 vs 132 antibiotic courses, respectively). In contrast, the average rural NP prescribed 152 (115%) more antibiotic courses relative to the average urban NP (284 vs 132 antibiotic courses). Across the seven Southeast states, the average rural physician prescribed 81–266 more antibiotic courses, and the average rural NP prescribed 170–268 more antibiotic courses (Figure 5 and Table S4). This regional pattern was attenuated after adjusting for the average-provider rate *per capita*. Specifically, in 44 of 47 states with rural counties, the average rural physician and NP prescribed more antibiotic courses per 100,000 population (median = 14, interquartile range [IQR] = 10–23 for physicians; median = 19, IQR = 13–31 for NPs). The three states where the average urban physician and NP prescribed more antibiotics compared to their rural counterparts were Montana, Vermont, and Wyoming (Figure S2 and Table S5). These rural–urban trends were consistent for the four commonly prescribed antibiotics evaluated for 2022: amoxicillin, azithromycin, cefdinir, and ciprofloxacin (Figures S3–S6 and Tables S6–S9).

## Discussion

This study reports the growing role of APCs in outpatient antibiotic prescribing in the United States over the past decade, with the proportion increasing from 1 in 7 prescriptions per capita in 2011 to 1 in 3 in 2022. This increase is consistent with prescribing patterns previously observed during 2011 through 2016,^6^ and was mostly driven by NPs, whose prescribing rate per capita almost tripled during our study period. By 2022, similar to physicians, prescribing rates by NPs were strongly associated with antibiotic prescribing overall when comparing states. The increase in NP prescribing rates was especially pronounced in the seven contiguous, higher-prescribing states in the Southeast region, particularly within rural counties, likely reflecting the growing role of NPs as primary care clinicians in rural areas.^2,24–27^

These findings are consistent with the relatively larger proportion of NPs (12%) that work in rural areas compared to physicians (7.2%), and they also mirror trends reported from other high-income countries^28^ and several U.S. states.^7,8^ In Kentucky, for example, from 2012 to 2017, per-capita antibiotic prescribing rates for children on Medicaid shifted from family physicians to NPs, who were the most common prescribers by 2017, accounting for almost 40% of prescriptions.^9^ Prescribing rates were highest in rural counties, consistent with the findings of our study.

This study also highlights the importance of understanding rural–urban differences in antibiotic prescribing for both physicians and APCs. Nationally, the average rural physician, NP, and PA wrote more prescriptions compared to their urban counterparts. While this rural–urban difference was especially pronounced in the seven highest prescribing states, that regional variation disappeared after accounting for population size, suggesting that the higher rate per average provider observed in the rural Southeast partly reflects the relatively larger rural population. Without data on diagnosis related to the prescription, the appropriateness of higher rural antibiotic prescribing could not be assessed. However, prior research of antibiotic prescribing in primary care suggests that greater total antibiotic prescribing is correlated with unnecessary prescribing,^29^ and that more inappropriate antibiotic prescribing occurs among rural (compared to urban) outpatients.^7,13,17–19,30–32^ Various factors for more inappropriate prescribing among rural outpatients have been cited, including challenges with applying watchful waiting with a “delayed prescription”^33^ (eg, due to greater difficulty of follow-up and providers’ concern for liability),^34^ decreased resource access for diagnostic or microbiologic testing,^35^ and patient-level factors (eg, health status,^34^ health literacy).^36^ However, we observed that the average rural provider prescribed more courses of broader spectrum, second-line antibiotics—such as fluoroquinolones, azithromycin, and cefdinir—suggesting that some prescribing may be inappropriate.^20^ While some studies suggest APCs prescribe a greater proportion of antibiotics in rural areas,^7–9^ investigations comparing the appropriateness of antibiotic prescribing between APCs and physicians have been mixed.^7,10–19^

Our findings have important implications for outpatient antibiotic stewardship for health departments, payors, and health systems. Identifying geographic differences in prescribing rates between provider groups can inform targeted stewardship efforts, which may include: (1) engaging APC professional societies to incorporate stewardship content in professional conferences and providing access to stewardship education; (2) developing and tailoring educational materials for APCs—recognizing that training models differ between NPs and PAs; and (3) ensuring APCs contribute to stewardship committees, quality improvement projects, and guideline development workgroups.^39^ Given the limited stewardship expertise and infrastructure in rural areas, state, local, and territorial health department stewardship experts can play an important role in engagement and support of rural clinicians to improve prescribing practices through quality improvement initiatives with rural health partners. Routine public health-supported activities in rural settings, including surveillance and outbreak response, can also integrate antibiotic stewardship communication and support.^37^ State Medicaid agencies and other payors can also play a critical role through quality improvement, data-driven audit and feedback, and financial incentives. With consolidation of healthcare delivery, health systems may play an increasingly important role in supporting stewardship in outpatient settings.^38^

This study has several limitations. As an ecological (county-level) analysis, our results may not reflect individual-level characteristics associated with prescribing. Comparison of antibiotic prescribing rates limited to 2011 and 2022 (because of availability of the provider type denominator) may be affected by other population-level changes, such as usage of non-pharmaceutical interventions (eg, masking and social distancing) related to the Coronavirus disease (COVID) 2019 pandemic. Per-capita rates were crude rates, that is unadjusted for age and sex. Per-provider rates of the “average” provider do not reflect provider variability in prescribing.^8,11,19^ Appropriateness of prescribing could not be assessed without an associated clinical diagnosis nor information on prescription duration. Finally, dispensed prescription data may not accurately represent actual consumption of antibiotics, as patient adherence varies.

In summary, our nationwide analysis of outpatient antibiotic prescribing in 2011 and 2022 showed that, during an era of declining per-capita antibiotic use, the proportion prescribed by APCs increased, especially for NPs, who accounted for about 1 in 4 antibiotic prescriptions nationwide in 2022. Trends suggest that much of this is due to the growing role of NPs as primary care clinicians in the United States, particularly in rural counties and the Southeast region. Understanding the differences in geography and provider type underlying outpatient antibiotic prescribing can guide antibiotic stewardship efforts in engaging APCs as antibiotic stewardship champions, aiming to improve the quality and consistency of patient care and prevent unnecessary adverse events associated with antibiotic use.

## Supporting information

10.1017/ash.2026.10346.sm001 Ali et al. supplementary material Ali et al. supplementary material

## Footnotes

1
See for example 45 C.F.R. part 46.102(l)(2), 21 C.F.R. part 56; 42 U.S.C. §241(d);  5 U.S.C. §552a; 44 U.S.C. §3501 et seq.

## Supplementary material

The supplementary material for this article can be found at https://doi.org/10.1017/ash.2026.10346.
