# Supplementary material for: The changing landscape of outpatient antibiotic prescriptions among advanced practice clinicians in the United States, 2011 and 2022
Source: Antimicrob Steward Healthc Epidemiol. 2026 Jun 1;6(1):e162. doi: 10.1017/ash.2026.10346 (PMC13227136; doi:10.1017/ash.2026.10346)
Supplement: Ali et al. supplementary material [file S2732494X26103465sup001.docx]

# Manuscript appendix

Contents

[Manuscript appendix 1](#_Toc201674266)

[Supplemental methods 2](#_Toc201674267)

[TABLE S1. Rates of outpatient antibiotic prescriptions per capita — United States, 2011 and 2022 3](#_Toc201674268)

[TABLE S2. Rates of outpatient antibiotic prescriptions by physicians, nurse practitioners, and physician assistants — United States, 2011 and 2022 6](#_Toc201674269)

[FIGURE S1. Outpatient antibiotic prescription rates per capita rate by state, 2011 versus 2022, stratified for physicians, nurse practitioners (NPs) and physician assistants (PAs). 11](#_Toc201674270)

[TABLE S3. Rates of rural and urban outpatient antibiotic prescriptions per capita by provider type by state — United States, 2022 12](#_Toc201674271)

[TABLE S4. Rates of rural and urban outpatient antibiotic prescriptions per provider by provider type and state — United States, 2022 15](#_Toc201674272)

[TABLE S5. Rates of rural and urban outpatient antibiotic prescriptions per provider per capita by provider type and state — United States, 2022 18](#_Toc201674273)

[FIGURE S2. Rural and urban outpatient antibiotic prescription rates per provider per capita for physicians and nurse practitioners — United States, 2022 20](#_Toc201674274)

[TABLE S6. Rates of rural and urban outpatient amoxicillin prescriptions by physicians and nurse practitioners by state — United States, 2022 21](#_Toc201674275)

[FIGURE S3. Choropleth maps of rural–urban rate difference of amoxicillin prescriptions by physicians and nurse practitioners by state — United States, 2022 24](#_Toc201674276)

[TABLE S7. Rates of rural and urban outpatient azithromycin prescriptions by physicians and nurse practitioners by state — United States, 2022 25](#_Toc201674277)

[FIGURE S4. Choropleth maps of rural–urban rate difference of azithromycin prescriptions by physicians and nurse practitioners by state — United States, 2022 28](#_Toc201674278)

[TABLE S8. Rates of rural and urban outpatient ciprofloxacin prescriptions by physicians and nurse practitioners by state — United States, 2022 29](#_Toc201674279)

[FIGURE S5. Choropleth maps of rural–urban rate difference of ciprofloxacin prescriptions by physicians and nurse practitioners by state — United States, 2022 32](#_Toc201674280)

[TABLE S9. Rates of rural and urban outpatient cefdinir prescriptions by physicians and nurse practitioners by state — United States, 2022 33](#_Toc201674281)

[FIGURE S6. Choropleth maps of rural–urban rate difference of cefdinir prescriptions by physicians and nurse practitioners by state — United States, 2022 36](#_Toc201674282)

## Supplemental methods

The following adjustments to denominators were made to calculate rates per capita and per provider.

For Connecticut’s population in 2022, we used 2021 county denominators (instead of planning regions from 2022) to align with NCHS in order to assign urban/rural status.

In cases where a provider’s ZIP code overlapped multiple counties, we used the county that had the highest proportion of business addresses among all business addresses in the entire ZIP to capture the most likely county in which the provider wrote the prescription (bus_ratio variable in dataset); a sensitivity analysis for all addresses in the ZIP (tot_ratio) was virtually identical.

## TABLE S1. Rates of outpatient antibiotic prescriptions per capita — United States, 2011 and 2022

|  | 2011 | |  | 2022 | |  | Change in rate | |
| --- | --- | --- | --- | --- | --- | --- | --- | --- |
|  | No. rx | Rate per 1000 popn |  | No. rx | Rate per 1000 popn |  | Absolute (per 1000 popn) | Relative (%) |
| TOTAL | 273,292,377 | 877 |  | 236,389,488 | 709 |  | -168 | -19 |
| Age category (years) |  |  |  |  |  |  |  |  |
| 0-9 | 45,225,589 | 1118 |  | 28,838,090 | 748 |  | -370 | -33 |
| 10-19 | 30,000,026 | 708 |  | 19,668,665 | 463 |  | -245 | -35 |
| 20-39 | 59,687,276 | 714 |  | 50,825,411 | 562 |  | -152 | -21 |
| 40-64 | 86,128,287 | 830 |  | 76,019,793 | 731 |  | -99 | -12 |
| 65+ | 46,039,993 | 1113 |  | 60,801,579 | 1052 |  | -61 | -5 |
| Sex |  |  |  |  |  |  |  |  |
| Female | 164,161,745 | 1037 |  | 144,901,726 | 863 |  | -174 | -17 |
| Male | 106,480,933 | 695 |  | 91,303,232 | 552 |  | -143 | -21 |
| County rurality |  |  |  |  |  |  |  |  |
| Rural |  |  |  | 32,614,047 | 707 |  |  |  |
| Urban |  |  |  | 203,631,227 | 709 |  |  |  |
| Region |  |  |  |  |  |  |  |  |
| Midwest | 63,378,675 | 944 |  | 49,465,642 | 719 |  | -225 | -24 |
| Northeast | 49,630,055 | 893 |  | 41,099,797 | 721 |  | -172 | -19 |
| South | 111,426,009 | 961 |  | 106,159,466 | 825 |  | -136 | -14 |
| West | 48,857,638 | 671 |  | 39,520,369 | 502 |  | -169 | -25 |
| State |  |  |  |  |  |  |  |  |
| Alabama | 5,539,607 | 1154 |  | 5,227,906 | 1030 |  | -124 | -11 |
| Alaska | 424,892 | 588 |  | 295,972 | 403 |  | -185 | -31 |
| Arizona | 5,256,558 | 812 |  | 4,666,303 | 634 |  | -178 | -22 |
| Arkansas | 3,031,470 | 1031 |  | 3,106,729 | 1020 |  | -11 | -1 |
| California | 23,699,864 | 630 |  | 18,336,061 | 470 |  | -160 | -25 |
| Colorado | 3,390,903 | 662 |  | 2,825,089 | 484 |  | -178 | -27 |
| Connecticut | 3,168,393 | 883 |  | 2,516,406 | 698 |  | -185 | -21 |
| Delaware | 930,051 | 1025 |  | 715,583 | 703 |  | -322 | -31 |
| District Of Columbia | 658,241 | 1062 |  | 502,229 | 748 |  | -314 | -30 |
| Florida | 14,860,908 | 780 |  | 17,371,926 | 781 |  | 1 | 0 |
| Georgia | 8,575,206 | 875 |  | 8,753,907 | 802 |  | -73 | -8 |
| Hawaii | 936,474 | 679 |  | 682,064 | 474 |  | -205 | -30 |
| Idaho | 1,171,701 | 740 |  | 1,095,680 | 565 |  | -175 | -24 |
| Illinois | 11,678,745 | 908 |  | 8,816,959 | 701 |  | -207 | -23 |
| Indiana | 6,898,647 | 1059 |  | 5,346,207 | 782 |  | -277 | -26 |
| Iowa | 2,870,113 | 936 |  | 2,433,590 | 760 |  | -176 | -19 |
| Kansas | 2,922,121 | 1018 |  | 2,359,299 | 803 |  | -215 | -21 |
| Kentucky | 5,772,102 | 1321 |  | 4,752,677 | 1053 |  | -268 | -20 |
| Louisiana | 5,463,419 | 1194 |  | 4,894,638 | 1066 |  | -128 | -11 |
| Maine | 1,020,769 | 768 |  | 791,426 | 571 |  | -197 | -26 |
| Maryland | 5,023,618 | 860 |  | 3,891,996 | 631 |  | -229 | -27 |
| Massachusetts | 5,568,588 | 842 |  | 4,338,214 | 621 |  | -221 | -26 |
| Michigan | 9,932,991 | 1005 |  | 7,577,104 | 755 |  | -250 | -25 |
| Minnesota | 3,988,710 | 746 |  | 2,985,647 | 522 |  | -224 | -30 |
| Mississippi | 3,532,113 | 1186 |  | 3,379,589 | 1149 |  | -37 | -3 |
| Missouri | 5,863,169 | 976 |  | 4,518,439 | 731 |  | -245 | -25 |
| Montana | 722,515 | 724 |  | 586,446 | 522 |  | -202 | -28 |
| Nebraska | 1,858,718 | 1010 |  | 1,621,234 | 824 |  | -186 | -18 |
| Nevada | 1,970,554 | 726 |  | 1,966,541 | 619 |  | -107 | -15 |
| New Hampshire | 953,890 | 723 |  | 858,521 | 615 |  | -108 | -15 |
| New Jersey | 8,515,899 | 965 |  | 6,938,791 | 749 |  | -216 | -22 |
| New Mexico | 1,485,669 | 714 |  | 1,304,378 | 617 |  | -97 | -14 |
| New York | 17,817,197 | 914 |  | 15,064,317 | 766 |  | -148 | -16 |
| North Carolina | 8,766,153 | 908 |  | 7,720,927 | 722 |  | -186 | -20 |
| North Dakota | 686,290 | 1002 |  | 505,566 | 649 |  | -353 | -35 |
| Ohio | 11,469,256 | 993 |  | 9,383,091 | 798 |  | -195 | -20 |
| Oklahoma | 3,453,313 | 912 |  | 3,246,824 | 808 |  | -104 | -11 |
| Oregon | 2,458,017 | 635 |  | 1,843,026 | 435 |  | -200 | -31 |
| Pennsylvania | 11,165,043 | 876 |  | 9,490,797 | 732 |  | -144 | -16 |
| Rhode Island | 983,847 | 934 |  | 762,269 | 697 |  | -237 | -25 |
| South Carolina | 4,398,964 | 942 |  | 4,332,660 | 820 |  | -122 | -13 |
| South Dakota | 761,793 | 925 |  | 656,790 | 722 |  | -203 | -22 |
| Tennessee | 7,975,753 | 1246 |  | 6,817,146 | 967 |  | -279 | -22 |
| Texas | 23,907,766 | 932 |  | 23,419,812 | 780 |  | -152 | -16 |
| Utah | 2,382,980 | 847 |  | 2,112,779 | 625 |  | -222 | -26 |
| Vermont | 436,429 | 696 |  | 339,057 | 524 |  | -172 | -25 |
| Virginia | 7,024,593 | 867 |  | 5,923,127 | 682 |  | -185 | -21 |
| Washington | 4,518,143 | 662 |  | 3,405,589 | 437 |  | -225 | -34 |
| West Virginia | 2,512,732 | 1354 |  | 2,101,791 | 1184 |  | -170 | -13 |
| Wisconsin | 4,448,124 | 780 |  | 3,261,714 | 554 |  | -226 | -29 |
| Wyoming | 439,368 | 774 |  | 400,440 | 689 |  | -85 | -11 |
| Provider type |  |  |  |  |  |  |  |  |
| Dentists | 22,967,915 | 74 |  | 25,189,848 | 76 |  | 2 | 3 |
| Nurse Practitioners | 20,204,165 | 65 |  | 53,669,745 | 161 |  | 96 | 148 |
| Other | 8,597,934 | 28 |  | 8,524,553 | 26 |  | -2 | -7 |
| Physician Assistants | 18,316,069 | 59 |  | 30,754,566 | 92 |  | 33 | 56 |
| Physicians | 195,641,942 | 628 |  | 114,975,223 | 345 |  | -283 | -45 |
| Unknown | 7,564,353 | 24 |  | 3,275,553 | 10 |  | -14 | -58 |
| Antibiotic category |  |  |  |  |  |  |  |  |
| Beta-lactams, increased activity | 22,050,704 | 71 |  | 28,700,394 | 86 |  | 15 | 21 |
| Cephalosporins | 36,575,915 | 117 |  | 35,965,264 | 108 |  | -9 | -8 |
| Fluoroquinolones | 31,573,548 | 101 |  | 14,753,618 | 44 |  | -57 | -56 |
| Lincosamides | 8,201,205 | 26 |  | 8,263,367 | 25 |  | -1 | -4 |
| Macrolides | 59,162,720 | 190 |  | 36,148,356 | 108 |  | -82 | -43 |
| Other | 546,936 | 2 |  | 732,638 | 2 |  | 0 | 0 |
| Penicillins | 61,093,327 | 196 |  | 53,152,882 | 159 |  | -37 | -19 |
| TMP-SMX | 20,791,531 | 67 |  | 14,719,909 | 44 |  | -23 | -34 |
| Tetracyclines | 21,157,539 | 68 |  | 27,121,737 | 81 |  | 13 | 19 |
| Urinary anti-infectives | 12,138,953 | 39 |  | 16,831,323 | 51 |  | 12 | 31 |
| Antibiotic (top 10 either year) |  |  |  |  |  |  |  |  |
| Amoxicillin | 52,905,191 | 170 |  | 49,775,962 | 149 |  | -21 | -12 |
| Amoxicillin-Clavulanic Acid | 22,050,704 | 71 |  | 28,700,394 | 86 |  | 15 | 21 |
| Azithromycin | 54,302,690 | 174 |  | 34,886,271 | 105 |  | -69 | -40 |
| Cefdinir | 9,403,707 | 30 |  | 11,003,634 | 33 |  | 3 | 10 |
| Cephalexin | 20,278,576 | 65 |  | 21,039,590 | 63 |  | -2 | -3 |
| Ciprofloxacin | 21,027,960 | 67 |  | 9,829,318 | 29 |  | -38 | -57 |
| Clindamycin | 8,198,126 | 26 |  | 8,217,944 | 25 |  | -1 | -4 |
| Doxycycline | 14,901,667 | 48 |  | 24,117,688 | 72 |  | 24 | 50 |
| Levofloxacin | 8,147,686 | 26 |  | 4,769,449 | 14 |  | -12 | -46 |
| Nitrofurantoin | 8,030,561 | 26 |  | 12,709,149 | 38 |  | 12 | 46 |
| Sulfamethoxazole-Trimethoprim | 20,446,680 | 66 |  | 14,505,019 | 44 |  | -22 | -33 |

Abbreviations: popn, population; rx, prescription. Data source: IQVIA Xponent®.

## TABLE S2. Rates of outpatient antibiotic prescriptions by physicians, nurse practitioners, and physician assistants — United States, 2011 and 2022

|  | Physicians | | | | | |  | Nurse practitioners | | | | |  | Physician assistants | | | | | | |
| --- | --- | --- | --- | --- | --- | --- | --- | --- | --- | --- | --- | --- | --- | --- | --- | --- | --- | --- | --- | --- |
|  | Rate per 1000 popn | | Change in rate | | | Rate per provider in 2022 |  | Rate per 1000 popn | | Change in rate | | Rate per provider in 2022 |  | Rate per 1000 popn | | | Change in rate | | Rate per provider in 2022 | |
|  | 2011 | 2022 | Absolute (per 1000 popn) | | Relative (%) |  |  | 2011 | 2022 | Absolute (per 1000 popn) | Relative (%) |  |  | 2011 | 2022 | | Absolute (per 1000 popn) | Relative (%) |  |  |
| TOTAL | 628 | 345 | -283 | | -45 | 138 |  | 65 | 161 | 96 | 148 | 150 |  | 59 | | 92 | 33 | 56 | 199 |  |
| Age group (years) |  |  |  | |  |  |  |  |  |  |  |  |  |  | |  |  |  |  |  |
| 0-9 | 876 | 408 | -468 | | -53 | 19 |  | 95 | 212 | 117 | 123 | 23 |  | 69 | | 86 | 17 | 25 | 21 |  |
| 10-19 | 520 | 225 | -295 | | -57 | 11 |  | 62 | 122 | 60 | 97 | 15 |  | 57 | | 71 | 14 | 25 | 20 |  |
| 20-39 | 483 | 240 | -243 | | -50 | 26 |  | 62 | 146 | 84 | 135 | 37 |  | 62 | | 93 | 31 | 50 | 54 |  |
| 40-64 | 572 | 342 | -230 | | -40 | 43 |  | 57 | 160 | 103 | 181 | 46 |  | 53 | | 94 | 41 | 77 | 63 |  |
| 65+ | 827 | 558 | -269 | | -33 | 39 |  | 53 | 181 | 128 | 242 | 29 |  | 48 | | 108 | 60 | 125 | 40 |  |
| Sex |  |  |  | |  |  |  |  |  |  |  |  |  |  | |  |  |  |  |  |
| Female | 742 | 417 | -325 | | -44 | 84 |  | 82 | 208 | 126 | 154 | 98 |  | 71 | | 115 | 44 | 62 | 125 |  |
| Male | 499 | 272 | -227 | | -45 | 54 |  | 45 | 113 | 68 | 151 | 52 |  | 44 | | 69 | 25 | 57 | 74 |  |
| County rurality |  |  |  | |  |  |  |  |  |  |  |  |  |  | |  |  |  |  |  |
| Rural |  | 271 |  | |  | 207 |  |  | 264 |  |  | 284 |  |  | | 91 |  |  | 289 |  |
| Urban |  | 357 |  | |  | 132 |  |  | 144 |  |  | 132 |  |  | | 92 |  |  | 189 |  |
| Region |  |  |  | |  |  |  |  |  |  |  |  |  |  | |  |  |  |  |  |
| Midwest | 683 | 355 | -328 | | -48 | 132 |  | 69 | 170 | 101 | 146 | 147 |  | 56 | | 83 | 27 | 48 | 192 |  |
| Northeast | 627 | 378 | -249 | | -40 | 117 |  | 63 | 107 | 44 | 70 | 92 |  | 64 | | 113 | 49 | 77 | 166 |  |
| South | 710 | 392 | -318 | | -45 | 175 |  | 73 | 227 | 154 | 211 | 196 |  | 49 | | 93 | 44 | 90 | 232 |  |
| West | 447 | 234 | -213 | | -48 | 103 |  | 50 | 85 | 35 | 70 | 105 |  | 73 | | 84 | 11 | 15 | 190 |  |
| State |  |  |  | |  |  |  |  |  |  |  |  |  |  | |  |  |  |  |  |
| Alabama | 993 | 563 | -430 | | -43 | 254 |  | 21 | 309 | 288 | 1371 | 266 |  | 7 | | 34 | 27 | 386 | 212 |  |
| Alaska | 320 | 189 | -131 | | -41 | 85 |  | 79 | 75 | -4 | -5 | 54 |  | 98 | | 76 | -22 | -22 | 83 |  |
| Arizona | 470 | 255 | -215 | | -46 | 126 |  | 99 | 163 | 64 | 65 | 145 |  | 125 | | 115 | -10 | -8 | 233 |  |
| Arkansas | 752 | 464 | -288 | | -38 | 229 |  | 107 | 379 | 272 | 254 | 295 |  | 27 | | 52 | 25 | 93 | 270 |  |
| California | 451 | 237 | -214 | | -47 | 99 |  | 28 | 57 | 29 | 104 | 96 |  | 54 | | 68 | 14 | 26 | 202 |  |
| Colorado | 410 | 209 | -201 | | -49 | 91 |  | 54 | 89 | 35 | 65 | 85 |  | 95 | | 101 | 6 | 6 | 140 |  |
| Connecticut | 609 | 354 | -255 | | -42 | 109 |  | 66 | 105 | 39 | 59 | 78 |  | 73 | | 120 | 47 | 64 | 154 |  |
| Delaware | 701 | 338 | -363 | | -52 | 137 |  | 105 | 158 | 53 | 50 | 117 |  | 90 | | 110 | 20 | 22 | 181 |  |
| District of Columbia | 726 | 404 | -322 | | -44 | 52 |  | 55 | 114 | 59 | 107 | 56 |  | 48 | | 96 | 48 | 100 | 116 |  |
| Florida | 638 | 403 | -235 | | -37 | 174 |  | 22 | 171 | 149 | 677 | 133 |  | 14 | | 83 | 69 | 493 | 209 |  |
| Georgia | 720 | 406 | -314 | | -44 | 199 |  | 24 | 206 | 182 | 758 | 198 |  | 22 | | 85 | 63 | 286 | 209 |  |
| Hawaii | 551 | 312 | -239 | | -43 | 119 |  | 11 | 49 | 38 | 345 | 74 |  | 21 | | 39 | 18 | 86 | 150 |  |
| Idaho | 419 | 199 | -220 | | -53 | 128 |  | 83 | 121 | 38 | 46 | 121 |  | 143 | | 159 | 16 | 11 | 240 |  |
| Illinois | 656 | 364 | -292 | | -45 | 127 |  | 58 | 149 | 91 | 157 | 152 |  | 50 | | 68 | 18 | 36 | 198 |  |
| Indiana | 756 | 366 | -390 | | -52 | 171 |  | 133 | 236 | 103 | 77 | 179 |  | 30 | | 59 | 29 | 97 | 200 |  |
| Iowa | 622 | 335 | -287 | | -46 | 163 |  | 99 | 232 | 133 | 134 | 194 |  | 108 | | 107 | -1 | -1 | 255 |  |
| Kansas | 707 | 382 | -325 | | -46 | 159 |  | 106 | 223 | 117 | 110 | 166 |  | 81 | | 94 | 13 | 16 | 210 |  |
| Kentucky | 923 | 439 | -484 | | -52 | 188 |  | 179 | 423 | 244 | 136 | 260 |  | 51 | | 73 | 22 | 43 | 251 |  |
| Louisiana | 918 | 526 | -392 | | -43 | 194 |  | 88 | 316 | 228 | 259 | 258 |  | 22 | | 91 | 69 | 314 | 293 |  |
| Maine | 443 | 247 | -196 | | -44 | 91 |  | 118 | 134 | 16 | 14 | 91 |  | 96 | | 110 | 14 | 15 | 148 |  |
| Maryland | 586 | 304 | -282 | | -48 | 104 |  | 67 | 125 | 58 | 87 | 107 |  | 67 | | 97 | 30 | 45 | 180 |  |
| Massachusetts | 536 | 291 | -245 | | -46 | 78 |  | 107 | 126 | 19 | 18 | 81 |  | 56 | | 89 | 33 | 59 | 142 |  |
| Michigan | 731 | 382 | -349 | | -48 | 124 |  | 37 | 117 | 80 | 216 | 124 |  | 69 | | 120 | 51 | 74 | 196 |  |
| Minnesota | 497 | 255 | -242 | | -49 | 97 |  | 77 | 105 | 28 | 36 | 99 |  | 81 | | 93 | 12 | 15 | 160 |  |
| Mississippi | 800 | 447 | -353 | | -44 | 214 |  | 222 | 554 | 332 | 150 | 328 |  | 5 | | 27 | 22 | 440 | 233 |  |
| Missouri | 818 | 374 | -444 | | -54 | 134 |  | 16 | 207 | 191 | 1194 | 193 |  | 2 | | 36 | 34 | 1700 | 206 |  |
| Montana | 395 | 207 | -188 | | -48 | 100 |  | 100 | 117 | 17 | 17 | 101 |  | 129 | | 120 | -9 | -7 | 165 |  |
| Nebraska | 682 | 396 | -286 | | -42 | 152 |  | 73 | 167 | 94 | 129 | 141 |  | 145 | | 157 | 12 | 8 | 223 |  |
| Nevada | 491 | 283 | -208 | | -42 | 163 |  | 47 | 132 | 85 | 181 | 155 |  | 75 | | 99 | 24 | 32 | 266 |  |
| New Hampshire | 421 | 249 | -172 | | -41 | 100 |  | 113 | 151 | 38 | 34 | 103 |  | 83 | | 118 | 35 | 42 | 182 |  |
| New Jersey | 762 | 460 | -302 | | -40 | 174 |  | 39 | 90 | 51 | 131 | 104 |  | 30 | | 75 | 45 | 150 | 217 |  |
| New Mexico | 403 | 228 | -175 | | -43 | 106 |  | 114 | 206 | 92 | 81 | 164 |  | 74 | | 80 | 6 | 8 | 188 |  |
| New York | 629 | 405 | -224 | | -36 | 118 |  | 61 | 97 | 36 | 59 | 82 |  | 76 | | 128 | 52 | 68 | 158 |  |
| North Carolina | 556 | 301 | -255 | | -46 | 137 |  | 90 | 163 | 73 | 81 | 154 |  | 139 | | 154 | 15 | 11 | 203 |  |
| North Dakota | 652 | 309 | -343 | | -53 | 129 |  | 137 | 188 | 51 | 37 | 134 |  | 101 | | 79 | -22 | -22 | 154 |  |
| Ohio | 749 | 389 | -360 | | -48 | 135 |  | 72 | 210 | 138 | 192 | 145 |  | 22 | | 73 | 51 | 232 | 198 |  |
| Oklahoma | 628 | 368 | -260 | | -41 | 181 |  | 68 | 222 | 154 | 226 | 246 |  | 107 | | 109 | 2 | 2 | 243 |  |
| Oregon | 403 | 198 | -205 | | -51 | 84 |  | 67 | 76 | 9 | 13 | 81 |  | 63 | | 80 | 17 | 27 | 148 |  |
| Pennsylvania | 632 | 371 | -261 | | -41 | 116 |  | 46 | 113 | 67 | 146 | 115 |  | 68 | | 128 | 60 | 88 | 177 |  |
| Rhode Island | 663 | 345 | -318 | | -48 | 101 |  | 71 | 137 | 66 | 93 | 108 |  | 57 | | 107 | 50 | 88 | 196 |  |
| South Carolina | 702 | 412 | -290 | | -41 | 184 |  | 87 | 207 | 120 | 138 | 197 |  | 42 | | 89 | 47 | 112 | 229 |  |
| South Dakota | 542 | 301 | -241 | | -44 | 136 |  | 95 | 188 | 93 | 98 | 145 |  | 187 | | 143 | -44 | -24 | 208 |  |
| Tennessee | 762 | 375 | -387 | | -51 | 170 |  | 236 | 370 | 134 | 57 | 214 |  | 77 | | 101 | 24 | 31 | 270 |  |
| Texas | 726 | 380 | -346 | | -48 | 199 |  | 40 | 201 | 161 | 403 | 227 |  | 39 | | 96 | 57 | 146 | 293 |  |
| Utah | 532 | 287 | -245 | | -46 | 159 |  | 79 | 111 | 32 | 41 | 115 |  | 115 | | 122 | 7 | 6 | 223 |  |
| Vermont | 434 | 219 | -215 | | -50 | 79 |  | 76 | 119 | 43 | 57 | 94 |  | 84 | | 98 | 14 | 17 | 163 |  |
| Virginia | 600 | 327 | -273 | | -45 | 141 |  | 87 | 157 | 70 | 80 | 152 |  | 57 | | 92 | 35 | 61 | 206 |  |
| Washington | 423 | 200 | -223 | | -53 | 83 |  | 63 | 74 | 11 | 17 | 77 |  | 79 | | 81 | 2 | 3 | 158 |  |
| West Virginia | 894 | 491 | -403 | | -45 | 174 |  | 119 | 358 | 239 | 201 | 253 |  | 164 | | 195 | 31 | 19 | 316 |  |
| Wisconsin | 522 | 279 | -243 | | -47 | 112 |  | 74 | 108 | 34 | 46 | 96 |  | 84 | | 84 | 0 | 0 | 148 |  |
| Wyoming | 440 | 271 | -169 | | -38 | 135 |  | 95 | 193 | 98 | 103 | 184 |  | 147 | | 129 | -18 | -12 | 248 |  |
| Antibiotic category |  |  |  | |  |  |  |  |  |  |  |  |  |  | |  |  |  |  |  |
| Beta-lactams, increased activity | 53 | 41 | -12 | -23 | | 16 |  | 6 | 24 | 18 | 300 | 22 |  | 5 | | 13 | 8 | 160 | 27 |  |
| Cephalosporins | 88 | 58 | -30 | -34 | | 23 |  | 8 | 24 | 16 | 200 | 22 |  | 8 | | 16 | 8 | 100 | 36 |  |
| Fluoroquinolones | 82 | 28 | -54 | -66 | | 11 |  | 6 | 9 | 3 | 50 | 8 |  | 6 | | 5 | -1 | -17 | 10 |  |
| Lincosamides | 12 | 9 | -3 | -25 | | 4 |  | 1 | 3 | 2 | 200 | 3 |  | 1 | | 2 | 1 | 100 | 5 |  |
| Macrolides | 145 | 56 | -89 | -61 | | 23 |  | 16 | 29 | 13 | 81 | 27 |  | 14 | | 13 | -1 | -7 | 28 |  |
| Other | 1 | 2 | 1 | 100 | | 1 |  | 0 | 0 | 0 |  | 0 |  | 0 | | 0 | 0 |  | 0 |  |
| Penicillins | 112 | 58 | -54 | -48 | | 23 |  | 14 | 29 | 15 | 107 | 27 |  | 11 | | 14 | 3 | 27 | 31 |  |
| TMP-SMX | 51 | 25 | -26 | -51 | | 10 |  | 6 | 10 | 4 | 67 | 10 |  | 5 | | 6 | 1 | 20 | 14 |  |
| Tetracyclines | 52 | 42 | -10 | -19 | | 17 |  | 4 | 18 | 14 | 350 | 17 |  | 6 | | 15 | 9 | 150 | 33 |  |
| Urinary anti-infectives | 31 | 27 | -4 | -13 | | 11 |  | 4 | 15 | 11 | 275 | 14 |  | 3 | | 7 | 4 | 133 | 16 |  |
| Antibiotic (top 10 either year) |  |  |  | |  |  |  |  |  |  |  |  |  |  | |  |  |  |  |  |
| Amoxicillin | 101 | 54 | -47 | | -47 | 22 |  | 12 | 27 | 15 | 125 | 25 |  | 9 | | 13 | 4 | 44 | 29 |  |
| Amoxicillin-Clavulanic Acid | 53 | 41 | -12 | | -23 | 16 |  | 6 | 24 | 18 | 300 | 22 |  | 5 | | 13 | 8 | 160 | 27 |  |
| Azithromycin | 133 | 54 | -79 | | -59 | 22 |  | 15 | 28 | 13 | 87 | 26 |  | 13 | | 13 | 0 | 0 | 28 |  |
| Cefdinir | 24 | 18 | -6 | | -25 | 7 |  | 3 | 10 | 7 | 233 | 9 |  | 2 | | 4 | 2 | 100 | 10 |  |
| Cephalexin | 46 | 33 | -13 | | -28 | 13 |  | 4 | 12 | 8 | 200 | 12 |  | 5 | | 10 | 5 | 100 | 22 |  |
| Ciprofloxacin | 55 | 18 | -37 | | -67 | 7 |  | 4 | 6 | 2 | 50 | 6 |  | 4 | | 3 | -1 | -25 | 7 |  |
| Clindamycin | 12 | 9 | -3 | | -25 | 4 |  | 1 | 3 | 2 | 200 | 3 |  | 1 | | 2 | 1 | 100 | 5 |  |
| Doxycycline | 36 | 37 | 1 | | 3 | 15 |  | 3 | 16 | 13 | 433 | 15 |  | 4 | | 13 | 9 | 225 | 29 |  |
| Levofloxacin | 21 | 9 | -12 | | -57 | 4 |  | 2 | 3 | 1 | 50 | 2 |  | 1 | | 1 | 0 | 0 | 3 |  |
| Nitrofurantoin | 21 | 20 | -1 | | -5 | 8 |  | 2 | 12 | 10 | 500 | 11 |  | 2 | | 5 | 3 | 150 | 12 |  |
| Sulfamethoxazole-Trimethoprim | 50 | 24 | -26 | | -52 | 10 |  | 6 | 10 | 4 | 67 | 10 |  | 5 | | 6 | 1 | 20 | 13 |  |

Abbreviation: popn, population. Data source: IQVIA Xponent®.

## FIGURE S1. Outpatient antibiotic prescription rates per capita rate by state, 2011 versus 2022, stratified for physicians, nurse practitioners (NPs) and physician assistants (PAs).


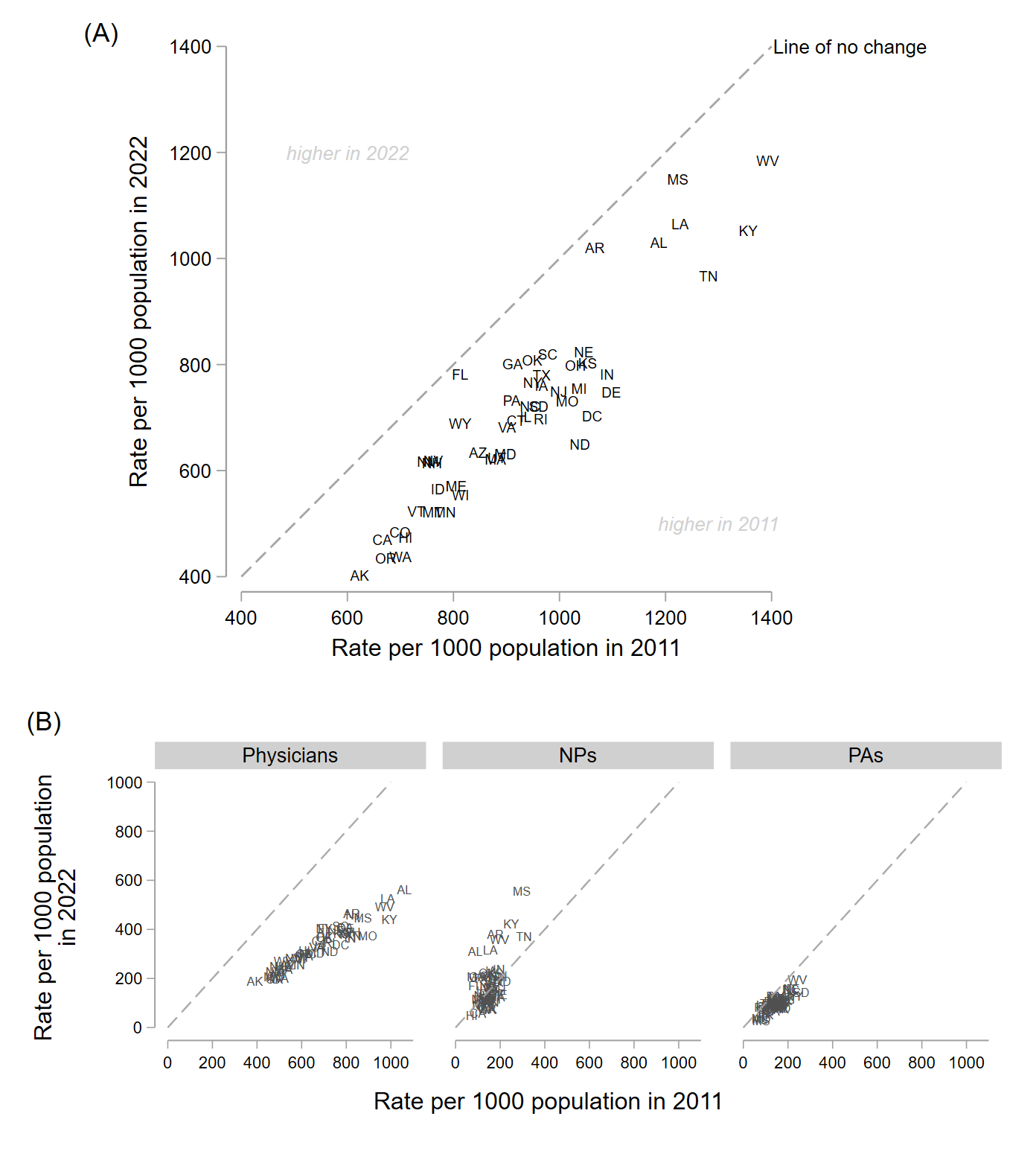


Data source: IQVIA Xponent®.

## TABLE S3. Rates of rural and urban outpatient antibiotic prescriptions per capita by provider type by state — United States, 2022

|  | **Physicians’ rate per 1000 popn** | | |  | **NP rate per 1000 popn** | | |  | **PA rate per 1000 popn** | | |
| --- | --- | --- | --- | --- | --- | --- | --- | --- | --- | --- | --- |
| **State** | **Rural** | **Urban** | **Difference** |  | **Rural** | **Urban** | **Difference** |  | **Rural** | **Urban** | **Difference** |
| Alabama | 479 | 587 | -108 |  | 405 | 281 | 124 |  | 16 | 39 | -23 |
| Alaska | 120 | 223 | -103 |  | 84 | 71 | 13 |  | 52 | 88 | -36 |
| Arizona | 154 | 260 | -106 |  | 110 | 166 | -56 |  | 89 | 117 | -28 |
| Arkansas | 378 | 513 | -135 |  | 429 | 351 | 78 |  | 46 | 56 | -10 |
| California | 147 | 239 | -92 |  | 80 | 56 | 24 |  | 58 | 69 | -11 |
| Colorado | 185 | 212 | -27 |  | 97 | 88 | 9 |  | 94 | 102 | -8 |
| Connecticut | 261 | 359 | -98 |  | 65 | 108 | -43 |  | 79 | 123 | -44 |
| Delaware |  | 338 |  |  |  | 158 |  |  |  | 110 |  |
| District Of Columbia |  | 404 |  |  |  | 114 |  |  |  | 96 |  |
| Florida | 197 | 409 | -212 |  | 255 | 169 | 86 |  | 54 | 84 | -30 |
| Georgia | 399 | 408 | -9 |  | 385 | 170 | 215 |  | 93 | 84 | 9 |
| Hawaii | 288 | 318 | -30 |  | 70 | 44 | 26 |  | 47 | 37 | 10 |
| Idaho | 176 | 210 | -34 |  | 119 | 122 | -3 |  | 158 | 160 | -2 |
| Illinois | 279 | 375 | -96 |  | 271 | 134 | 137 |  | 66 | 68 | -2 |
| Indiana | 243 | 399 | -156 |  | 244 | 234 | 10 |  | 34 | 66 | -32 |
| Iowa | 239 | 397 | -158 |  | 272 | 207 | 65 |  | 76 | 128 | -52 |
| Kansas | 312 | 414 | -102 |  | 253 | 210 | 43 |  | 102 | 91 | 11 |
| Kentucky | 397 | 469 | -72 |  | 586 | 312 | 274 |  | 98 | 57 | 41 |
| Louisiana | 391 | 551 | -160 |  | 442 | 293 | 149 |  | 69 | 95 | -26 |
| Maine | 229 | 259 | -30 |  | 138 | 132 | 6 |  | 92 | 122 | -30 |
| Maryland | 350 | 303 | 47 |  | 274 | 122 | 152 |  | 80 | 97 | -17 |
| Massachusetts | 101 | 294 | -193 |  | 79 | 126 | -47 |  | 48 | 89 | -41 |
| Michigan | 214 | 419 | -205 |  | 135 | 114 | 21 |  | 118 | 120 | -2 |
| Minnesota | 187 | 274 | -87 |  | 140 | 95 | 45 |  | 96 | 93 | 3 |
| Mississippi | 360 | 543 | -183 |  | 651 | 448 | 203 |  | 20 | 35 | -15 |
| Missouri | 265 | 410 | -145 |  | 281 | 183 | 98 |  | 33 | 37 | -4 |
| Montana | 173 | 271 | -98 |  | 117 | 116 | 1 |  | 103 | 154 | -51 |
| Nebraska | 299 | 445 | -146 |  | 202 | 149 | 53 |  | 188 | 142 | 46 |
| Nevada | 145 | 297 | -152 |  | 92 | 136 | -44 |  | 122 | 97 | 25 |
| New Hampshire | 276 | 234 | 42 |  | 178 | 134 | 44 |  | 95 | 131 | -36 |
| New Jersey |  | 460 |  |  |  | 90 |  |  |  | 75 |  |
| New Mexico | 225 | 230 | -5 |  | 258 | 181 | 77 |  | 60 | 90 | -30 |
| New York | 236 | 417 | -181 |  | 151 | 94 | 57 |  | 152 | 126 | 26 |
| North Carolina | 260 | 311 | -51 |  | 213 | 150 | 63 |  | 159 | 153 | 6 |
| North Dakota | 207 | 405 | -198 |  | 208 | 169 | 39 |  | 86 | 72 | 14 |
| Ohio | 290 | 414 | -124 |  | 272 | 194 | 78 |  | 68 | 75 | -7 |
| Oklahoma | 289 | 406 | -117 |  | 297 | 186 | 111 |  | 90 | 119 | -29 |
| Oregon | 192 | 200 | -8 |  | 111 | 69 | 42 |  | 94 | 77 | 17 |
| Pennsylvania | 294 | 381 | -87 |  | 135 | 110 | 25 |  | 174 | 122 | 52 |
| Rhode Island |  | 345 |  |  |  | 137 |  |  |  | 107 |  |
| South Carolina | 329 | 426 | -97 |  | 208 | 207 | 1 |  | 76 | 91 | -15 |
| South Dakota | 218 | 382 | -164 |  | 190 | 187 | 3 |  | 128 | 157 | -29 |
| Tennessee | 265 | 407 | -142 |  | 531 | 324 | 207 |  | 98 | 102 | -4 |
| Texas | 261 | 393 | -132 |  | 255 | 195 | 60 |  | 80 | 98 | -18 |
| Utah | 251 | 291 | -40 |  | 113 | 111 | 2 |  | 169 | 117 | 52 |
| Vermont | 189 | 274 | -85 |  | 126 | 105 | 21 |  | 101 | 91 | 10 |
| Virginia | 276 | 334 | -58 |  | 323 | 135 | 188 |  | 74 | 95 | -21 |
| Washington | 131 | 207 | -76 |  | 73 | 74 | -1 |  | 87 | 80 | 7 |
| West Virginia | 403 | 543 | -140 |  | 385 | 341 | 44 |  | 244 | 166 | 78 |
| Wisconsin | 180 | 314 | -134 |  | 108 | 108 | 0 |  | 70 | 89 | -19 |
| Wyoming | 219 | 387 | -168 |  | 168 | 247 | -79 |  | 122 | 146 | -24 |
| UNITED STATES | 271 | 357 | -86 |  | 264 | 144 | 120 |  | 91 | 92 | -1 |

Abbreviations: NP, nurse practitioner; PA, physician assistant; popn, population. Data source: IQVIA Xponent®.

## TABLE S4. Rates of rural and urban outpatient antibiotic prescriptions per provider by provider type and state — United States, 2022

|  | **Physicians’ rate per provider** | | |  | **NP rate per provider** | | |  | **PA rate per provider** | | |
| --- | --- | --- | --- | --- | --- | --- | --- | --- | --- | --- | --- |
| **State** | **Rural** | **Urban** | **Difference** |  | **Rural** | **Urban** | **Difference** |  | **Rural** | **Urban** | **Difference** |
| Alabama | 494 | 228 | 266 |  | 483 | 224 | 259 |  | 347 | 202 | 145 |
| Alaska | 74 | 88 | -14 |  | 62 | 50 | 12 |  | 77 | 85 | -8 |
| Arizona | 133 | 126 | 7 |  | 143 | 145 | -2 |  | 265 | 232 | 33 |
| Arkansas | 354 | 199 | 155 |  | 429 | 242 | 187 |  | 444 | 228 | 216 |
| California | 100 | 99 | 1 |  | 124 | 95 | 29 |  | 160 | 203 | -43 |
| Colorado | 112 | 89 | 23 |  | 117 | 82 | 35 |  | 164 | 137 | 27 |
| Connecticut | 166 | 107 | 59 |  | 90 | 78 | 12 |  | 215 | 153 | 62 |
| Delaware |  | 137 |  |  |  | 117 |  |  |  | 181 |  |
| District Of Columbia |  | 53 |  |  |  | 56 |  |  |  | 116 |  |
| Florida | 204 | 174 | 30 |  | 297 | 130 | 167 |  | 383 | 207 | 176 |
| Georgia | 354 | 183 | 171 |  | 375 | 163 | 212 |  | 378 | 191 | 187 |
| Hawaii | 147 | 115 | 32 |  | 118 | 65 | 53 |  | 209 | 138 | 71 |
| Idaho | 154 | 119 | 35 |  | 181 | 105 | 76 |  | 349 | 209 | 140 |
| Illinois | 235 | 122 | 113 |  | 299 | 135 | 164 |  | 326 | 189 | 137 |
| Indiana | 230 | 164 | 66 |  | 280 | 162 | 118 |  | 305 | 191 | 114 |
| Iowa | 200 | 152 | 48 |  | 262 | 158 | 104 |  | 281 | 246 | 35 |
| Kansas | 226 | 145 | 81 |  | 244 | 142 | 102 |  | 243 | 196 | 47 |
| Kentucky | 285 | 157 | 128 |  | 401 | 179 | 222 |  | 420 | 170 | 250 |
| Louisiana | 362 | 183 | 179 |  | 449 | 231 | 218 |  | 535 | 277 | 258 |
| Maine | 108 | 84 | 24 |  | 112 | 81 | 31 |  | 173 | 138 | 35 |
| Maryland | 204 | 103 | 101 |  | 221 | 104 | 117 |  | 230 | 179 | 51 |
| Massachusetts | 84 | 78 | 6 |  | 74 | 81 | -7 |  | 131 | 143 | -12 |
| Michigan | 151 | 121 | 30 |  | 187 | 114 | 73 |  | 254 | 186 | 68 |
| Minnesota | 145 | 91 | 54 |  | 168 | 84 | 84 |  | 242 | 146 | 96 |
| Mississippi | 268 | 187 | 81 |  | 421 | 242 | 179 |  | 313 | 201 | 112 |
| Missouri | 224 | 123 | 101 |  | 341 | 159 | 182 |  | 332 | 186 | 146 |
| Montana | 111 | 89 | 22 |  | 109 | 88 | 21 |  | 173 | 156 | 17 |
| Nebraska | 214 | 138 | 76 |  | 219 | 113 | 106 |  | 299 | 191 | 108 |
| Nevada | 162 | 163 | -1 |  | 191 | 153 | 38 |  | 425 | 253 | 172 |
| New Hampshire | 80 | 121 | -41 |  | 107 | 100 | 7 |  | 144 | 204 | -60 |
| New Jersey |  | 174 |  |  |  | 104 |  |  |  | 217 |  |
| New Mexico | 172 | 89 | 83 |  | 254 | 132 | 122 |  | 251 | 174 | 77 |
| New York | 151 | 117 | 34 |  | 171 | 78 | 93 |  | 273 | 152 | 121 |
| North Carolina | 190 | 130 | 60 |  | 257 | 134 | 123 |  | 311 | 186 | 125 |
| North Dakota | 175 | 115 | 60 |  | 188 | 100 | 88 |  | 222 | 116 | 106 |
| Ohio | 211 | 127 | 84 |  | 261 | 126 | 135 |  | 317 | 183 | 134 |
| Oklahoma | 240 | 168 | 72 |  | 352 | 199 | 153 |  | 309 | 226 | 83 |
| Oregon | 115 | 80 | 35 |  | 125 | 73 | 52 |  | 221 | 137 | 84 |
| Pennsylvania | 188 | 111 | 77 |  | 224 | 107 | 117 |  | 304 | 165 | 139 |
| Rhode Island |  | 101 |  |  |  | 108 |  |  |  | 196 |  |
| South Carolina | 236 | 179 | 57 |  | 240 | 191 | 49 |  | 337 | 220 | 117 |
| South Dakota | 167 | 124 | 43 |  | 217 | 110 | 107 |  | 251 | 185 | 66 |
| Tennessee | 280 | 158 | 122 |  | 441 | 173 | 268 |  | 410 | 247 | 163 |
| Texas | 316 | 194 | 122 |  | 423 | 213 | 210 |  | 500 | 283 | 217 |
| Utah | 246 | 153 | 93 |  | 198 | 110 | 88 |  | 408 | 207 | 201 |
| Vermont | 102 | 61 | 41 |  | 104 | 81 | 23 |  | 196 | 121 | 75 |
| Virginia | 221 | 136 | 85 |  | 340 | 129 | 211 |  | 377 | 197 | 180 |
| Washington | 115 | 82 | 33 |  | 106 | 75 | 31 |  | 210 | 154 | 56 |
| West Virginia | 243 | 154 | 89 |  | 377 | 207 | 170 |  | 473 | 245 | 228 |
| Wisconsin | 128 | 110 | 18 |  | 142 | 86 | 56 |  | 190 | 139 | 51 |
| Wyoming | 127 | 146 | -19 |  | 179 | 192 | -13 |  | 251 | 242 | 9 |
| UNITED STATES | 207 | 132 | 75 |  | 284 | 132 | 152 |  | 289 | 189 | 100 |

Abbreviations: NP, nurse practitioner; PA, physician assistant; popn, population. Data source: IQVIA Xponent®.

## TABLE S5. Rates of rural and urban outpatient antibiotic prescriptions per provider per capita by provider type and state — United States, 2022

|  | **Physicians’ rate per provider per 100,000 popn** | | |  | **NP rate per provider per 100,000 popn** | | |  | | **PA rate per provider per 100,000 popn** | | | |  |
| --- | --- | --- | --- | --- | --- | --- | --- | --- | --- | --- | --- | --- | --- | --- |
| **State** | **Rural** | **Urban** | **Difference** |  | **Rural** | **Urban** | **Difference** | |  | | **Rural** | **Urban** | **Difference** | |
| Alabama | 43.208 | 5.813 | 37.396 |  | 42.282 | 5.701 | 36.581 | |  | | 30.394 | 5.146 | 25.248 | |
| Alaska | 31.025 | 17.709 | 13.316 |  | 26.272 | 10.146 | 16.126 | |  | | 32.215 | 17.180 | 15.034 | |
| Arizona | 38.949 | 1.789 | 37.160 |  | 41.811 | 2.072 | 39.739 | |  | | 77.682 | 3.309 | 74.373 | |
| Arkansas | 32.149 | 10.247 | 21.901 |  | 39.047 | 12.449 | 26.598 | |  | | 40.386 | 11.723 | 28.663 | |
| California | 11.751 | 0.259 | 11.492 |  | 14.588 | 0.249 | 14.340 | |  | | 18.830 | 0.533 | 18.297 | |
| Colorado | 15.682 | 1.745 | 13.937 |  | 16.304 | 1.599 | 14.705 | |  | | 22.866 | 2.683 | 20.183 | |
| Connecticut | 89.868 | 3.137 | 86.731 |  | 48.677 | 2.276 | 46.401 | |  | | 115.994 | 4.460 | 111.533 | |
| Delaware |  | 13.491 |  |  |  | 11.528 |  | |  | |  | 17.806 |  | |
| District of Columbia |  | 7.871 |  |  |  | 8.348 |  | |  | |  | 17.306 |  | |
| Florida | 28.254 | 0.807 | 27.448 |  | 41.184 | 0.602 | 40.582 | |  | | 53.052 | 0.961 | 52.090 | |
| Georgia | 19.419 | 2.014 | 17.404 |  | 20.569 | 1.797 | 18.772 | |  | | 20.754 | 2.096 | 18.658 | |
| Hawaii | 52.302 | 9.877 | 42.425 |  | 42.057 | 5.571 | 36.486 | |  | | 74.457 | 11.860 | 62.597 | |
| Idaho | 24.453 | 9.131 | 15.323 |  | 28.648 | 8.000 | 20.648 | |  | | 55.287 | 15.958 | 39.329 | |
| Illinois | 16.585 | 1.095 | 15.490 |  | 21.083 | 1.209 | 19.874 | |  | | 22.985 | 1.694 | 21.291 | |
| Indiana | 15.722 | 3.058 | 12.664 |  | 19.109 | 3.023 | 16.087 | |  | | 20.805 | 3.551 | 17.255 | |
| Iowa | 15.875 | 7.825 | 8.050 |  | 20.807 | 8.163 | 12.644 | |  | | 22.271 | 12.680 | 9.592 | |
| Kansas | 24.835 | 7.154 | 17.681 |  | 26.821 | 6.988 | 19.834 | |  | | 26.694 | 9.666 | 17.028 | |
| Kentucky | 15.515 | 5.875 | 9.639 |  | 21.838 | 6.694 | 15.144 | |  | | 22.860 | 6.347 | 16.513 | |
| Louisiana | 50.428 | 4.724 | 45.703 |  | 62.638 | 5.952 | 56.685 | |  | | 74.558 | 7.143 | 67.415 | |
| Maine | 19.322 | 10.126 | 9.196 |  | 20.136 | 9.736 | 10.399 | |  | | 31.012 | 16.620 | 14.392 | |
| Maryland | 133.957 | 1.715 | 132.242 |  | 145.154 | 1.727 | 143.426 | |  | | 151.537 | 2.976 | 148.561 | |
| Massachusetts | 79.300 | 1.128 | 78.172 |  | 70.033 | 1.184 | 68.849 | |  | | 123.831 | 2.073 | 121.758 | |
| Michigan | 8.296 | 1.476 | 6.820 |  | 10.282 | 1.386 | 8.896 | |  | | 13.987 | 2.266 | 11.721 | |
| Minnesota | 11.534 | 2.041 | 9.493 |  | 13.320 | 1.896 | 11.424 | |  | | 19.155 | 3.265 | 15.890 | |
| Mississippi | 17.344 | 13.409 | 3.935 |  | 27.259 | 17.309 | 9.949 | |  | | 20.287 | 14.411 | 5.876 | |
| Missouri | 14.764 | 2.643 | 12.121 |  | 22.518 | 3.402 | 19.116 | |  | | 21.868 | 3.988 | 17.880 | |
| Montana | 15.108 | 22.826 | -7.718 |  | 14.818 | 22.742 | -7.924 | |  | | 23.533 | 40.350 | -16.817 | |
| Nebraska | 32.462 | 10.575 | 21.887 |  | 33.280 | 8.641 | 24.638 | |  | | 45.428 | 14.600 | 30.828 | |
| Nevada | 54.856 | 5.649 | 49.207 |  | 64.676 | 5.299 | 59.377 | |  | | 143.842 | 8.790 | 135.051 | |
| New Hampshire | 15.437 | 13.779 | 1.658 |  | 20.788 | 11.335 | 9.453 | |  | | 27.888 | 23.228 | 4.660 | |
| New Jersey |  | 1.876 |  |  |  | 1.119 |  | |  | |  | 2.343 |  | |
| New Mexico | 24.753 | 6.297 | 18.457 |  | 36.562 | 9.278 | 27.284 | |  | | 36.187 | 12.274 | 23.913 | |
| New York | 11.156 | 0.639 | 10.517 |  | 12.594 | 0.423 | 12.170 | |  | | 20.118 | 0.828 | 19.290 | |
| North Carolina | 8.794 | 1.518 | 7.276 |  | 11.853 | 1.574 | 10.279 | |  | | 14.377 | 2.178 | 12.199 | |
| North Dakota | 46.490 | 28.431 | 18.060 |  | 50.133 | 24.858 | 25.275 | |  | | 58.985 | 28.619 | 30.365 | |
| Ohio | 9.061 | 1.346 | 7.715 |  | 11.204 | 1.334 | 9.870 | |  | | 13.591 | 1.942 | 11.649 | |
| Oklahoma | 18.321 | 6.184 | 12.137 |  | 26.849 | 7.351 | 19.498 | |  | | 23.614 | 8.331 | 15.283 | |
| Oregon | 16.557 | 2.261 | 14.295 |  | 17.975 | 2.057 | 15.917 | |  | | 31.677 | 3.872 | 27.804 | |
| Pennsylvania | 13.174 | 0.965 | 12.209 |  | 15.722 | 0.924 | 14.798 | |  | | 21.331 | 1.426 | 19.905 | |
| Rhode Island |  | 9.219 |  |  |  | 9.905 |  | |  | |  | 17.927 |  | |
| South Carolina | 32.440 | 3.931 | 28.509 |  | 33.022 | 4.193 | 28.829 | |  | | 46.403 | 4.830 | 41.574 | |
| South Dakota | 37.206 | 26.911 | 10.294 |  | 48.371 | 23.791 | 24.580 | |  | | 55.825 | 40.109 | 15.715 | |
| Tennessee | 17.941 | 2.883 | 15.058 |  | 28.306 | 3.144 | 25.162 | |  | | 26.324 | 4.493 | 21.831 | |
| Texas | 10.387 | 0.718 | 9.669 |  | 13.900 | 0.789 | 13.111 | |  | | 16.404 | 1.048 | 15.356 | |
| Utah | 70.825 | 5.054 | 65.771 |  | 56.952 | 3.625 | 53.327 | |  | | 117.256 | 6.825 | 110.431 | |
| Vermont | 24.389 | 26.671 | -2.282 |  | 24.794 | 35.533 | -10.739 | |  | | 46.758 | 53.167 | -6.408 | |
| Virginia | 21.393 | 1.778 | 19.615 |  | 32.843 | 1.680 | 31.162 | |  | | 36.423 | 2.573 | 33.850 | |
| Washington | 14.800 | 1.164 | 13.636 |  | 13.598 | 1.074 | 12.524 | |  | | 26.990 | 2.196 | 24.794 | |
| West Virginia | 36.779 | 13.852 | 22.928 |  | 56.921 | 18.613 | 38.309 | |  | | 71.469 | 21.970 | 49.500 | |
| Wisconsin | 8.308 | 2.521 | 5.787 |  | 9.225 | 1.975 | 7.250 | |  | | 12.373 | 3.197 | 9.176 | |
| Wyoming | 31.661 | 81.100 | -49.439 |  | 44.660 | 106.666 | -62.006 | |  | | 62.555 | 134.127 | -71.571 | |
| UNITED STATES | 0.449 | 0.046 | 0.403 |  | 0.616 | 0.046 | 0.570 | |  | | 0.626 | 0.066 | 0.560 | |

Abbreviations: NP, nurse practitioner; PA, physician assistant; popn, population. Data source: IQVIA Xponent®.

## FIGURE S2. Rural and urban outpatient antibiotic prescription rates per provider per capita for physicians and nurse practitioners — United States, 2022


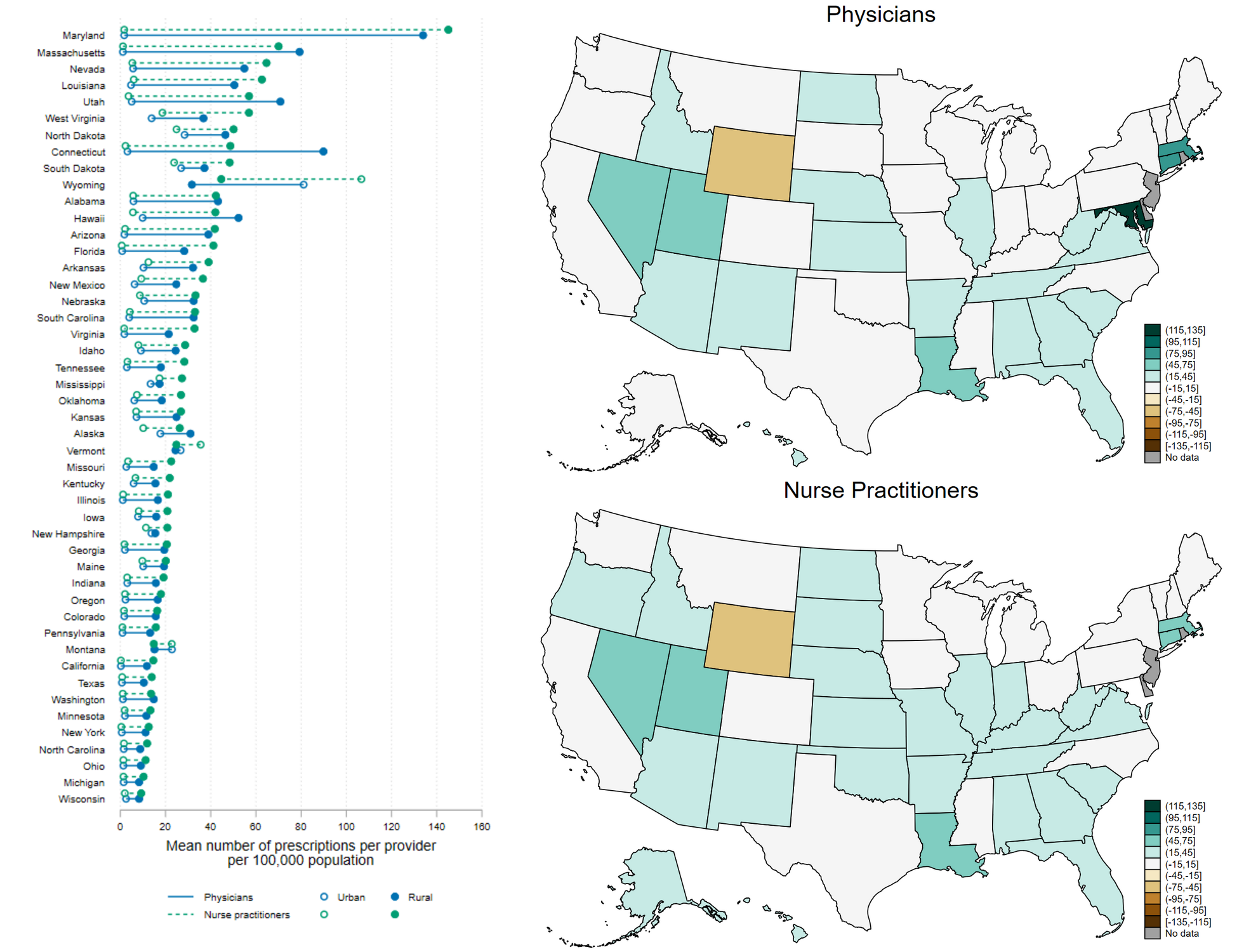

FIGURE S2. Rural and urban prescription rates per provider per capita (per 100,000 population) for physicians and nurse practitioners, by state. (A) Dumbbell plot sorted by rural nurse-practitioner rate. (B) Choropleth maps of rural–urban rate difference. Data source: IQVIA Xponent®.

## TABLE S6. Rates of rural and urban outpatient amoxicillin prescriptions by physicians and nurse practitioners by state — United States, 2022

|  | **Rate per 1000 popn** | | | | | | |  | **Rate per provider** | | | | | | |
| --- | --- | --- | --- | --- | --- | --- | --- | --- | --- | --- | --- | --- | --- | --- | --- |
|  | **Physicians** | | |  | **NP** | | |  | **Physicians** | | |  | **NP** | | |
| **State** | **Rural** | **Urban** | **Difference** |  | **Rural** | **Urban** | **Difference** |  | **Rural** | **Urban** | **Difference** |  | **Rural** | **Urban** | **Difference** |
| Alabama | 67 | 84 | -17 |  | 67 | 38 | 29 |  | 69 | 33 | 36 |  | 80 | 30 | 50 |
| Alaska | 13 | 33 | -20 |  | 11 | 9 | 2 |  | 8 | 13 | -5 |  | 8 | 6 | 2 |
| Arizona | 16 | 38 | -22 |  | 15 | 24 | -9 |  | 14 | 19 | -5 |  | 19 | 21 | -2 |
| Arkansas | 50 | 81 | -31 |  | 70 | 63 | 7 |  | 47 | 31 | 16 |  | 70 | 44 | 26 |
| California | 20 | 35 | -15 |  | 12 | 9 | 3 |  | 14 | 14 | 0 |  | 19 | 14 | 5 |
| Colorado | 26 | 33 | -7 |  | 16 | 14 | 2 |  | 16 | 14 | 2 |  | 19 | 13 | 6 |
| Connecticut | 52 | 61 | -9 |  | 9 | 16 | -7 |  | 33 | 18 | 15 |  | 12 | 12 | 0 |
| Delaware |  | 64 |  |  |  | 32 |  |  |  | 26 |  |  |  | 24 |  |
| District Of Columbia |  | 55 |  |  |  | 13 |  |  |  | 7 |  |  |  | 7 |  |
| Florida | 30 | 60 | -30 |  | 38 | 24 | 14 |  | 31 | 25 | 6 |  | 44 | 19 | 25 |
| Georgia | 66 | 71 | -5 |  | 71 | 31 | 40 |  | 58 | 32 | 26 |  | 70 | 30 | 40 |
| Hawaii | 30 | 45 | -15 |  | 7 | 5 | 2 |  | 15 | 16 | -1 |  | 13 | 8 | 5 |
| Idaho | 23 | 32 | -9 |  | 19 | 18 | 1 |  | 20 | 18 | 2 |  | 28 | 16 | 12 |
| Illinois | 39 | 64 | -25 |  | 55 | 25 | 30 |  | 33 | 21 | 12 |  | 60 | 25 | 35 |
| Indiana | 36 | 59 | -23 |  | 47 | 40 | 7 |  | 34 | 24 | 10 |  | 54 | 27 | 27 |
| Iowa | 37 | 67 | -30 |  | 52 | 39 | 13 |  | 31 | 26 | 5 |  | 50 | 30 | 20 |
| Kansas | 41 | 59 | -18 |  | 46 | 36 | 10 |  | 30 | 21 | 9 |  | 44 | 25 | 19 |
| Kentucky | 64 | 79 | -15 |  | 107 | 58 | 49 |  | 46 | 27 | 19 |  | 73 | 34 | 39 |
| Louisiana | 43 | 76 | -33 |  | 68 | 51 | 17 |  | 40 | 25 | 15 |  | 69 | 40 | 29 |
| Maine | 29 | 37 | -8 |  | 16 | 18 | -2 |  | 14 | 12 | 2 |  | 13 | 11 | 2 |
| Maryland | 52 | 51 | 1 |  | 47 | 20 | 27 |  | 30 | 18 | 12 |  | 38 | 17 | 21 |
| Massachusetts | 14 | 46 | -32 |  | 9 | 20 | -11 |  | 12 | 12 | 0 |  | 9 | 13 | -4 |
| Michigan | 29 | 66 | -37 |  | 22 | 20 | 2 |  | 20 | 19 | 1 |  | 30 | 20 | 10 |
| Minnesota | 31 | 48 | -17 |  | 26 | 17 | 9 |  | 25 | 16 | 9 |  | 31 | 15 | 16 |
| Mississippi | 43 | 65 | -22 |  | 95 | 69 | 26 |  | 32 | 22 | 10 |  | 62 | 37 | 25 |
| Missouri | 37 | 65 | -28 |  | 49 | 34 | 15 |  | 31 | 20 | 11 |  | 60 | 29 | 31 |
| Montana | 21 | 41 | -20 |  | 15 | 15 | 0 |  | 14 | 13 | 1 |  | 14 | 12 | 2 |
| Nebraska | 41 | 76 | -35 |  | 38 | 27 | 11 |  | 29 | 23 | 6 |  | 42 | 20 | 22 |
| Nevada | 27 | 47 | -20 |  | 13 | 20 | -7 |  | 31 | 26 | 5 |  | 27 | 23 | 4 |
| New Hampshire | 34 | 43 | -9 |  | 24 | 19 | 5 |  | 10 | 22 | -12 |  | 14 | 14 | 0 |
| New Jersey |  | 79 |  |  |  | 14 |  |  |  | 30 |  |  |  | 16 |  |
| New Mexico | 30 | 33 | -3 |  | 38 | 31 | 7 |  | 23 | 13 | 10 |  | 37 | 23 | 14 |
| New York | 37 | 69 | -32 |  | 22 | 14 | 8 |  | 24 | 19 | 5 |  | 25 | 12 | 13 |
| North Carolina | 37 | 47 | -10 |  | 34 | 23 | 11 |  | 27 | 20 | 7 |  | 41 | 21 | 20 |
| North Dakota | 33 | 68 | -35 |  | 34 | 25 | 9 |  | 28 | 19 | 9 |  | 31 | 15 | 16 |
| Ohio | 37 | 64 | -27 |  | 45 | 33 | 12 |  | 27 | 20 | 7 |  | 43 | 21 | 22 |
| Oklahoma | 37 | 57 | -20 |  | 56 | 37 | 19 |  | 30 | 24 | 6 |  | 66 | 40 | 26 |
| Oregon | 21 | 31 | -10 |  | 15 | 8 | 7 |  | 12 | 12 | 0 |  | 17 | 9 | 8 |
| Pennsylvania | 48 | 66 | -18 |  | 23 | 19 | 4 |  | 30 | 19 | 11 |  | 37 | 19 | 18 |
| Rhode Island |  | 54 |  |  |  | 19 |  |  |  | 16 |  |  |  | 15 |  |
| South Carolina | 48 | 68 | -20 |  | 41 | 33 | 8 |  | 35 | 29 | 6 |  | 47 | 30 | 17 |
| South Dakota | 39 | 74 | -35 |  | 37 | 33 | 4 |  | 30 | 24 | 6 |  | 42 | 19 | 23 |
| Tennessee | 31 | 61 | -30 |  | 86 | 51 | 35 |  | 33 | 24 | 9 |  | 72 | 27 | 45 |
| Texas | 38 | 64 | -26 |  | 49 | 38 | 11 |  | 45 | 31 | 14 |  | 82 | 41 | 41 |
| Utah | 49 | 58 | -9 |  | 17 | 21 | -4 |  | 48 | 30 | 18 |  | 30 | 21 | 9 |
| Vermont | 29 | 50 | -21 |  | 15 | 16 | -1 |  | 16 | 11 | 5 |  | 13 | 12 | 1 |
| Virginia | 43 | 54 | -11 |  | 54 | 22 | 32 |  | 34 | 22 | 12 |  | 57 | 21 | 36 |
| Washington | 15 | 30 | -15 |  | 9 | 9 | 0 |  | 13 | 12 | 1 |  | 14 | 9 | 5 |
| West Virginia | 48 | 82 | -34 |  | 68 | 51 | 17 |  | 29 | 23 | 6 |  | 66 | 31 | 35 |
| Wisconsin | 29 | 54 | -25 |  | 16 | 17 | -1 |  | 20 | 19 | 1 |  | 20 | 13 | 7 |
| Wyoming | 31 | 54 | -23 |  | 27 | 40 | -13 |  | 18 | 20 | -2 |  | 29 | 31 | -2 |

Abbreviations: NP, nurse practitioner; popn, population. Data source: IQVIA Xponent®.

## FIGURE S3. Choropleth maps of rural–urban rate difference of amoxicillin prescriptions by physicians and nurse practitioners by state — United States, 2022


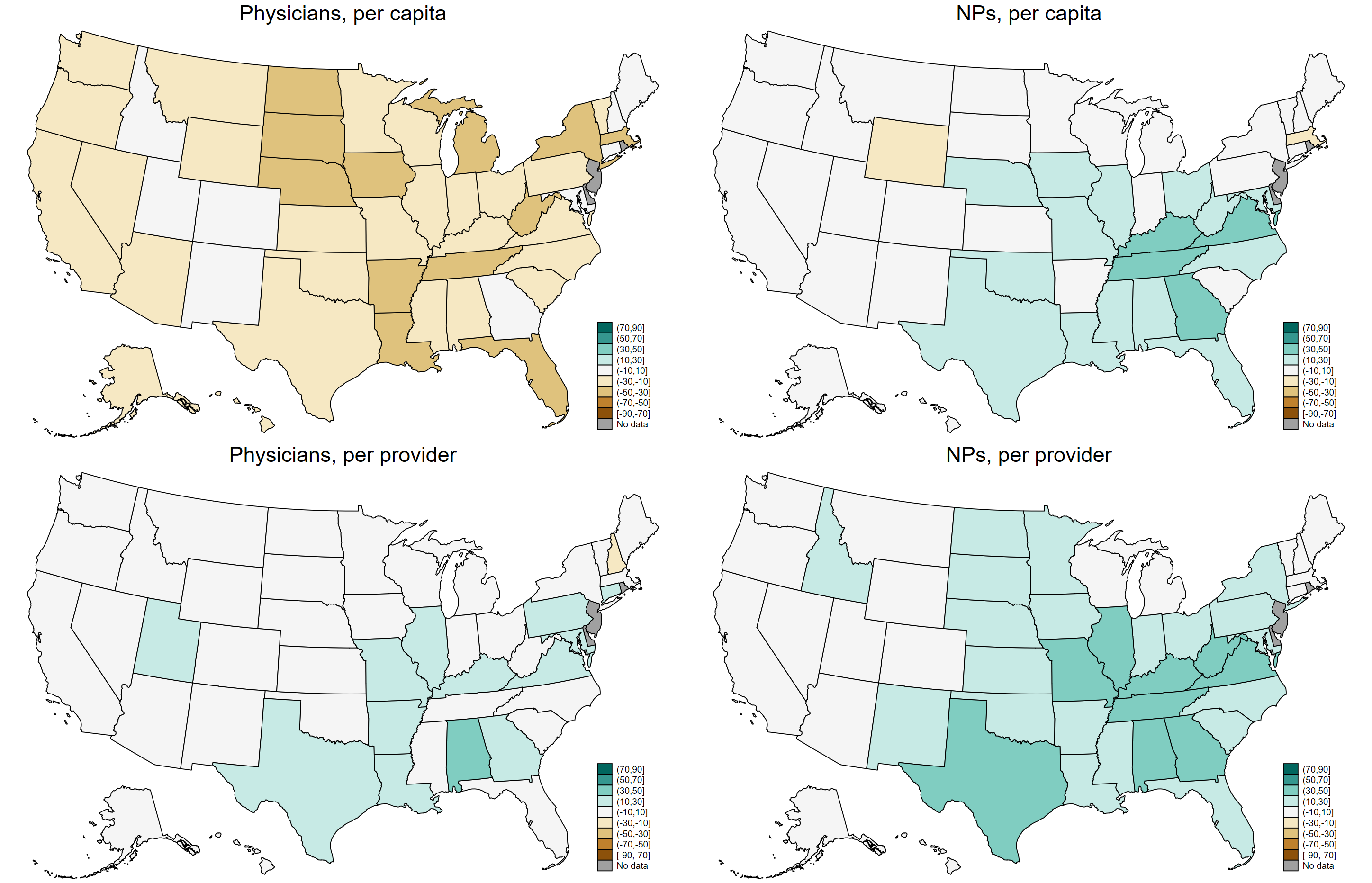


FIGURE S3. Choropleth maps of rural--urban rate difference of amoxcillin by physicians and nurse practitioners (NPs) by state. Data source: IQVIA Xponent®.

## TABLE S7. Rates of rural and urban outpatient azithromycin prescriptions by physicians and nurse practitioners by state — United States, 2022

|  | **Rate per 1000 popn** | | | | | | |  | **Rate per provider** | | | | | | |
| --- | --- | --- | --- | --- | --- | --- | --- | --- | --- | --- | --- | --- | --- | --- | --- |
|  | **Physicians** | | |  | **NP** | | |  | **Physicians** | | |  | **NP** | | |
| **State** | **Rural** | **Urban** | **Difference** |  | **Rural** | **Urban** | **Difference** |  | **Rural** | **Urban** | **Difference** |  | **Rural** | **Urban** | **Difference** |
| Alabama | 112 | 95 | 17 |  | 106 | 66 | 40 |  | 116 | 37 | 79 |  | 127 | 53 | 74 |
| Alaska | 16 | 28 | -12 |  | 12 | 10 | 2 |  | 10 | 11 | -1 |  | 9 | 7 | 2 |
| Arizona | 28 | 39 | -11 |  | 20 | 29 | -9 |  | 24 | 19 | 5 |  | 26 | 25 | 1 |
| Arkansas | 86 | 80 | 6 |  | 104 | 68 | 36 |  | 80 | 31 | 49 |  | 104 | 47 | 57 |
| California | 19 | 41 | -22 |  | 12 | 10 | 2 |  | 13 | 17 | -4 |  | 19 | 17 | 2 |
| Colorado | 24 | 25 | -1 |  | 15 | 11 | 4 |  | 14 | 11 | 3 |  | 18 | 11 | 7 |
| Connecticut | 42 | 54 | -12 |  | 13 | 14 | -1 |  | 27 | 16 | 11 |  | 18 | 10 | 8 |
| Delaware |  | 45 |  |  |  | 22 |  |  |  | 18 |  |  |  | 17 |  |
| District Of Columbia |  | 47 |  |  |  | 15 |  |  |  | 6 |  |  |  | 7 |  |
| Florida | 41 | 73 | -32 |  | 57 | 34 | 23 |  | 42 | 31 | 11 |  | 66 | 26 | 40 |
| Georgia | 74 | 64 | 10 |  | 85 | 31 | 54 |  | 66 | 29 | 37 |  | 82 | 29 | 53 |
| Hawaii | 46 | 55 | -9 |  | 11 | 6 | 5 |  | 23 | 20 | 3 |  | 18 | 9 | 9 |
| Idaho | 25 | 26 | -1 |  | 20 | 19 | 1 |  | 22 | 15 | 7 |  | 31 | 16 | 15 |
| Illinois | 52 | 58 | -6 |  | 46 | 19 | 27 |  | 43 | 19 | 24 |  | 50 | 19 | 31 |
| Indiana | 36 | 52 | -16 |  | 39 | 32 | 7 |  | 34 | 21 | 13 |  | 45 | 23 | 22 |
| Iowa | 36 | 55 | -19 |  | 43 | 26 | 17 |  | 30 | 21 | 9 |  | 41 | 20 | 21 |
| Kansas | 53 | 51 | 2 |  | 42 | 22 | 20 |  | 39 | 18 | 21 |  | 41 | 15 | 26 |
| Kentucky | 65 | 63 | 2 |  | 126 | 48 | 78 |  | 47 | 21 | 26 |  | 86 | 28 | 58 |
| Louisiana | 95 | 87 | 8 |  | 122 | 61 | 61 |  | 88 | 29 | 59 |  | 124 | 48 | 76 |
| Maine | 28 | 28 | 0 |  | 17 | 13 | 4 |  | 13 | 9 | 4 |  | 14 | 8 | 6 |
| Maryland | 52 | 44 | 8 |  | 39 | 18 | 21 |  | 30 | 15 | 15 |  | 32 | 16 | 16 |
| Massachusetts | 10 | 37 | -27 |  | 8 | 15 | -7 |  | 8 | 10 | -2 |  | 8 | 10 | -2 |
| Michigan | 31 | 68 | -37 |  | 23 | 18 | 5 |  | 22 | 20 | 2 |  | 31 | 18 | 13 |
| Minnesota | 23 | 31 | -8 |  | 17 | 12 | 5 |  | 18 | 10 | 8 |  | 21 | 10 | 11 |
| Mississippi | 68 | 93 | -25 |  | 188 | 117 | 71 |  | 51 | 32 | 19 |  | 122 | 63 | 59 |
| Missouri | 46 | 55 | -9 |  | 50 | 23 | 27 |  | 39 | 16 | 23 |  | 61 | 20 | 41 |
| Montana | 27 | 27 | 0 |  | 17 | 16 | 1 |  | 17 | 9 | 8 |  | 16 | 12 | 4 |
| Nebraska | 45 | 49 | -4 |  | 34 | 16 | 18 |  | 33 | 15 | 18 |  | 37 | 12 | 25 |
| Nevada | 18 | 52 | -34 |  | 18 | 24 | -6 |  | 20 | 28 | -8 |  | 38 | 27 | 11 |
| New Hampshire | 31 | 23 | 8 |  | 19 | 14 | 5 |  | 9 | 12 | -3 |  | 11 | 11 | 0 |
| New Jersey |  | 93 |  |  |  | 18 |  |  |  | 35 |  |  |  | 21 |  |
| New Mexico | 53 | 29 | 24 |  | 70 | 28 | 42 |  | 40 | 11 | 29 |  | 69 | 21 | 48 |
| New York | 37 | 77 | -40 |  | 25 | 15 | 10 |  | 24 | 22 | 2 |  | 28 | 12 | 16 |
| North Carolina | 38 | 36 | 2 |  | 38 | 20 | 18 |  | 27 | 15 | 12 |  | 46 | 18 | 28 |
| North Dakota | 28 | 39 | -11 |  | 26 | 18 | 8 |  | 24 | 11 | 13 |  | 24 | 11 | 13 |
| Ohio | 48 | 58 | -10 |  | 47 | 26 | 21 |  | 35 | 18 | 17 |  | 45 | 17 | 28 |
| Oklahoma | 53 | 66 | -13 |  | 65 | 33 | 32 |  | 44 | 27 | 17 |  | 76 | 35 | 41 |
| Oregon | 25 | 19 | 6 |  | 16 | 7 | 9 |  | 15 | 8 | 7 |  | 18 | 7 | 11 |
| Pennsylvania | 46 | 54 | -8 |  | 21 | 15 | 6 |  | 30 | 16 | 14 |  | 35 | 14 | 21 |
| Rhode Island |  | 55 |  |  |  | 20 |  |  |  | 16 |  |  |  | 16 |  |
| South Carolina | 59 | 63 | -4 |  | 39 | 34 | 5 |  | 42 | 26 | 16 |  | 46 | 32 | 14 |
| South Dakota | 29 | 45 | -16 |  | 28 | 18 | 10 |  | 22 | 15 | 7 |  | 32 | 11 | 21 |
| Tennessee | 47 | 53 | -6 |  | 119 | 57 | 62 |  | 50 | 21 | 29 |  | 98 | 30 | 68 |
| Texas | 57 | 66 | -9 |  | 63 | 41 | 22 |  | 69 | 32 | 37 |  | 104 | 45 | 59 |
| Utah | 28 | 29 | -1 |  | 19 | 14 | 5 |  | 27 | 15 | 12 |  | 33 | 14 | 19 |
| Vermont | 21 | 30 | -9 |  | 12 | 11 | 1 |  | 11 | 7 | 4 |  | 10 | 9 | 1 |
| Virginia | 48 | 47 | 1 |  | 68 | 19 | 49 |  | 38 | 19 | 19 |  | 71 | 18 | 53 |
| Washington | 18 | 22 | -4 |  | 10 | 8 | 2 |  | 15 | 9 | 6 |  | 15 | 8 | 7 |
| West Virginia | 68 | 72 | -4 |  | 70 | 53 | 17 |  | 41 | 21 | 20 |  | 68 | 32 | 36 |
| Wisconsin | 21 | 33 | -12 |  | 12 | 11 | 1 |  | 15 | 12 | 3 |  | 16 | 9 | 7 |
| Wyoming | 35 | 51 | -16 |  | 25 | 30 | -5 |  | 20 | 19 | 1 |  | 26 | 24 | 2 |

Abbreviations: NP, nurse practitioner; popn, population. Data source: IQVIA Xponent®.

## FIGURE S4. Choropleth maps of rural–urban rate difference of azithromycin prescriptions by physicians and nurse practitioners by state — United States, 2022


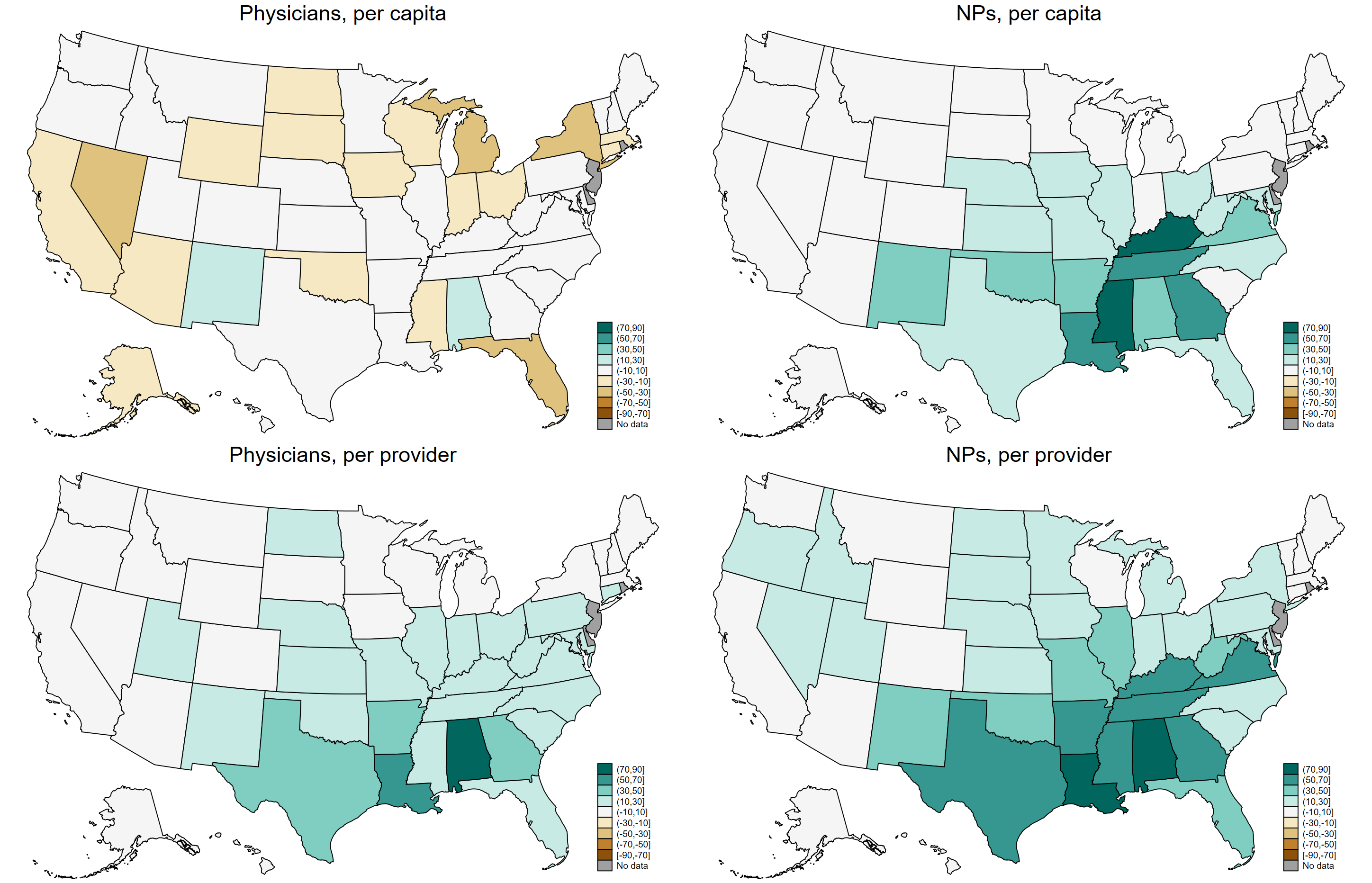


FIGURE S4. Choropleth maps of rural--urban rate difference of azithromycin by physicians and nurse practitioners (NPs) by state. Data source: IQVIA Xponent®.

## TABLE S8. Rates of rural and urban outpatient ciprofloxacin prescriptions by physicians and nurse practitioners by state — United States, 2022

|  | **Rate per 1000 popn** | | | | | | |  | **Rate per provider** | | | | | | |
| --- | --- | --- | --- | --- | --- | --- | --- | --- | --- | --- | --- | --- | --- | --- | --- |
|  | **Physicians** | | |  | **NP** | | |  | **Physicians** | | |  | **NP** | | |
| **State** | **Rural** | **Urban** | **Difference** |  | **Rural** | **Urban** | **Difference** |  | **Rural** | **Urban** | **Difference** |  | **Rural** | **Urban** | **Difference** |
| Alabama | 27 | 28 | -1 |  | 14 | 9 | 5 |  | 28 | 11 | 17 |  | 17 | 7 | 10 |
| Alaska | 6 | 9 | -3 |  | 3 | 3 | 0 |  | 4 | 3 | 1 |  | 2 | 2 | 0 |
| Arizona | 9 | 17 | -8 |  | 5 | 7 | -2 |  | 8 | 8 | 0 |  | 7 | 6 | 1 |
| Arkansas | 22 | 26 | -4 |  | 19 | 11 | 8 |  | 21 | 10 | 11 |  | 19 | 8 | 11 |
| California | 7 | 15 | -8 |  | 3 | 2 | 1 |  | 5 | 6 | -1 |  | 5 | 4 | 1 |
| Colorado | 8 | 9 | -1 |  | 4 | 3 | 1 |  | 5 | 4 | 1 |  | 5 | 2 | 3 |
| Connecticut | 12 | 16 | -4 |  | 3 | 4 | -1 |  | 8 | 5 | 3 |  | 4 | 3 | 1 |
| Delaware |  | 17 |  |  |  | 6 |  |  |  | 7 |  |  |  | 4 |  |
| District Of Columbia |  | 21 |  |  |  | 4 |  |  |  | 3 |  |  |  | 2 |  |
| Florida | 15 | 30 | -15 |  | 13 | 9 | 4 |  | 15 | 13 | 2 |  | 15 | 7 | 8 |
| Georgia | 24 | 22 | 2 |  | 15 | 7 | 8 |  | 21 | 10 | 11 |  | 15 | 6 | 9 |
| Hawaii | 17 | 24 | -7 |  | 4 | 2 | 2 |  | 9 | 9 | 0 |  | 6 | 3 | 3 |
| Idaho | 7 | 10 | -3 |  | 4 | 4 | 0 |  | 6 | 6 | 0 |  | 6 | 3 | 3 |
| Illinois | 13 | 17 | -4 |  | 8 | 4 | 4 |  | 11 | 6 | 5 |  | 9 | 4 | 5 |
| Indiana | 11 | 18 | -7 |  | 7 | 7 | 0 |  | 11 | 8 | 3 |  | 8 | 5 | 3 |
| Iowa | 11 | 16 | -5 |  | 8 | 5 | 3 |  | 10 | 6 | 4 |  | 8 | 4 | 4 |
| Kansas | 17 | 19 | -2 |  | 11 | 5 | 6 |  | 12 | 7 | 5 |  | 10 | 4 | 6 |
| Kentucky | 19 | 21 | -2 |  | 18 | 9 | 9 |  | 13 | 7 | 6 |  | 12 | 5 | 7 |
| Louisiana | 24 | 33 | -9 |  | 16 | 11 | 5 |  | 22 | 11 | 11 |  | 16 | 9 | 7 |
| Maine | 11 | 12 | -1 |  | 6 | 5 | 1 |  | 5 | 4 | 1 |  | 5 | 3 | 2 |
| Maryland | 20 | 15 | 5 |  | 11 | 5 | 6 |  | 12 | 5 | 7 |  | 8 | 4 | 4 |
| Massachusetts | 3 | 12 | -9 |  | 2 | 4 | -2 |  | 3 | 3 | 0 |  | 2 | 2 | 0 |
| Michigan | 13 | 23 | -10 |  | 5 | 4 | 1 |  | 9 | 7 | 2 |  | 7 | 4 | 3 |
| Minnesota | 9 | 11 | -2 |  | 5 | 2 | 3 |  | 7 | 4 | 3 |  | 6 | 2 | 4 |
| Mississippi | 23 | 31 | -8 |  | 23 | 15 | 8 |  | 17 | 11 | 6 |  | 15 | 8 | 7 |
| Missouri | 14 | 22 | -8 |  | 9 | 6 | 3 |  | 12 | 6 | 6 |  | 12 | 6 | 6 |
| Montana | 8 | 12 | -4 |  | 4 | 3 | 1 |  | 5 | 4 | 1 |  | 4 | 2 | 2 |
| Nebraska | 13 | 16 | -3 |  | 6 | 4 | 2 |  | 9 | 5 | 4 |  | 7 | 3 | 4 |
| Nevada | 7 | 17 | -10 |  | 4 | 6 | -2 |  | 8 | 9 | -1 |  | 9 | 7 | 2 |
| New Hampshire | 12 | 10 | 2 |  | 5 | 4 | 1 |  | 3 | 5 | -2 |  | p3 | 3 | 0 |
| New Jersey |  | 21 |  |  |  | 3 |  |  |  | 8 |  |  |  | 4 |  |
| New Mexico | 11 | 14 | -3 |  | 10 | 6 | 4 |  | 8 | 5 | 3 |  | 10 | 5 | 5 |
| New York | 13 | 22 | -9 |  | 6 | 4 | 2 |  | 8 | 6 | 2 |  | 6 | 3 | 3 |
| North Carolina | 16 | 17 | -1 |  | 9 | 6 | 3 |  | 12 | 7 | 5 |  | 11 | 5 | 6 |
| North Dakota | 10 | 20 | -10 |  | 7 | 6 | 1 |  | 9 | 6 | 3 |  | 7 | 4 | 3 |
| Ohio | 17 | 21 | -4 |  | 8 | 6 | 2 |  | 12 | 6 | 6 |  | 8 | 4 | 4 |
| Oklahoma | 17 | 23 | -6 |  | 11 | 6 | 5 |  | 14 | 9 | 5 |  | 13 | 6 | 7 |
| Oregon | 11 | 10 | 1 |  | 5 | 3 | 2 |  | 7 | 4 | 3 |  | 6 | 3 | 3 |
| Pennsylvania | 17 | 18 | -1 |  | 5 | 4 | 1 |  | 11 | 5 | 6 |  | 8 | 3 | 5 |
| Rhode Island |  | 17 |  |  |  | 5 |  |  |  | 5 |  |  |  | 4 |  |
| South Carolina | 21 | 24 | -3 |  | 10 | 9 | 1 |  | 15 | 10 | 5 |  | 11 | 8 | 3 |
| South Dakota | 12 | 17 | -5 |  | 7 | 6 | 1 |  | 9 | 5 | 4 |  | 8 | 3 | 5 |
| Tennessee | 15 | 23 | -8 |  | 18 | 12 | 6 |  | 16 | 9 | 7 |  | 15 | 6 | 9 |
| Texas | 17 | 21 | -4 |  | 10 | 7 | 3 |  | 21 | 10 | 11 |  | 17 | 8 | 9 |
| Utah | 11 | 11 | 0 |  | 4 | 4 | 0 |  | 10 | 6 | 4 |  | 8 | 4 | 4 |
| Vermont | 9 | 11 | -2 |  | 4 | 3 | 1 |  | 5 | 2 | 3 |  | 3 | 2 | 1 |
| Virginia | 14 | 15 | -1 |  | 13 | 5 | 8 |  | 11 | 6 | 5 |  | 14 | 5 | 9 |
| Washington | 7 | 9 | -2 |  | 3 | 2 | 1 |  | 6 | 4 | 2 |  | 4 | 2 | 2 |
| West Virginia | 23 | 27 | -4 |  | 13 | 11 | 2 |  | 14 | 8 | 6 |  | 13 | 7 | 6 |
| Wisconsin | 9 | 14 | -5 |  | 4 | 4 | 0 |  | 6 | 5 | 1 |  | 5 | 3 | 2 |
| Wyoming | 9 | 14 | -5 |  | 5 | 5 | 0 |  | 5 | 5 | 0 |  | 5 | 4 | 1 |

Abbreviations: NP, nurse practitioner; popn, population. Data source: IQVIA Xponent®.

## FIGURE S5. Choropleth maps of rural–urban rate difference of ciprofloxacin prescriptions by physicians and nurse practitioners by state — United States, 2022


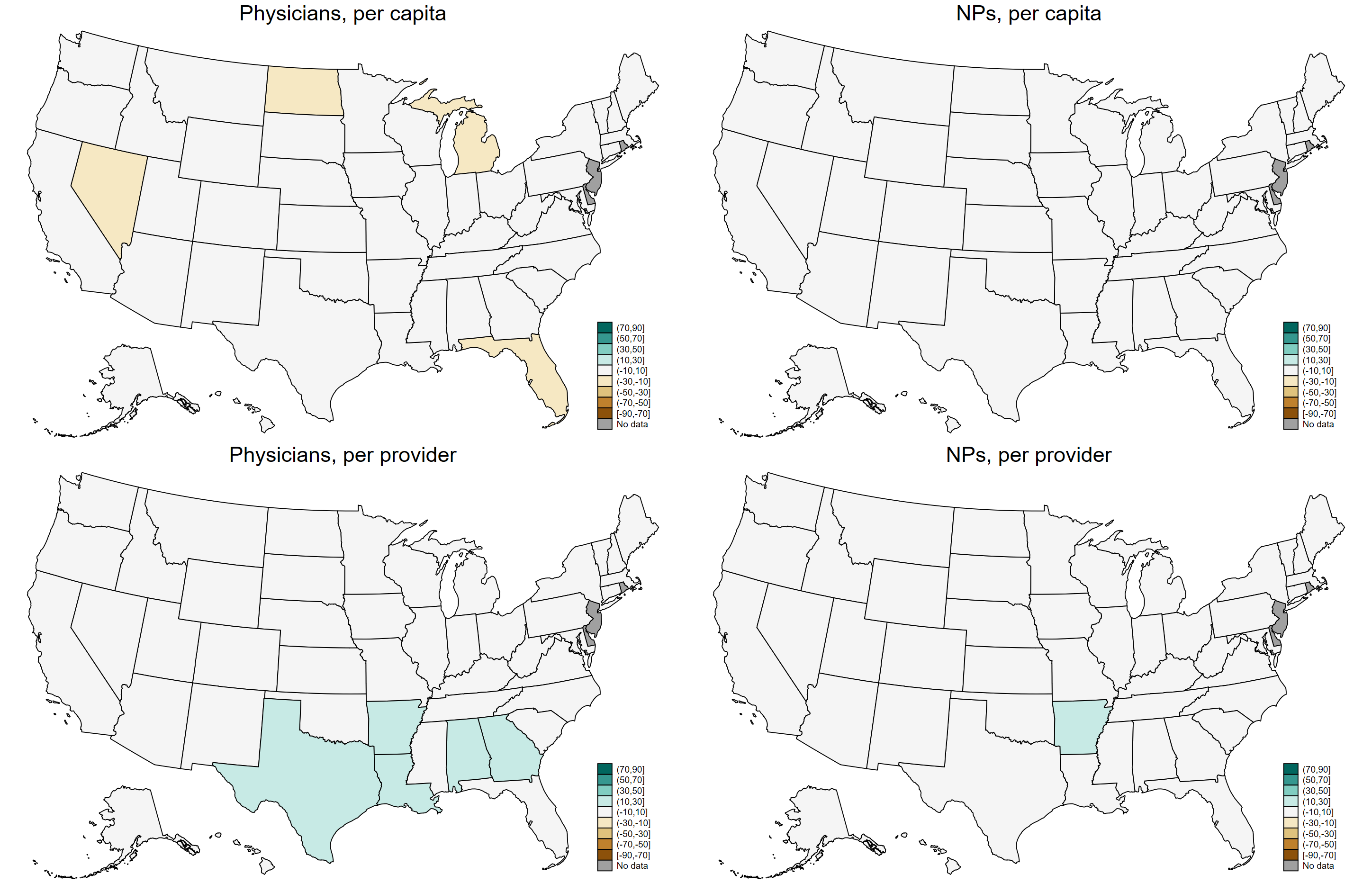


FIGURE S5. Choropleth maps of rural--urban rate difference of ciprofloxacin by physicians and nurse practitioners (NPs) by state. Abbreviations: NP, nurse practitioner; popn, population. Data source: IQVIA Xponent®.

## TABLE S9. Rates of rural and urban outpatient cefdinir prescriptions by physicians and nurse practitioners by state — United States, 2022

|  | **Rate per 1000 popn** | | | | | | |  | **Rate per provider** | | | | | | |
| --- | --- | --- | --- | --- | --- | --- | --- | --- | --- | --- | --- | --- | --- | --- | --- |
|  | **Physicians** | | |  | **NP** | | |  | **Physicians** | | |  | **NP** | | |
| **State** | **Rural** | **Urban** | **Difference** |  | **Rural** | **Urban** | **Difference** |  | **Rural** | **Urban** | **Difference** |  | **Rural** | **Urban** | **Difference** |
| Alabama | 42 | 48 | -6 |  | 46 | 26 | 20 |  | 43 | 19 | 24 |  | 55 | 21 | 34 |
| Alaska | 4 | 11 | -7 |  | 4 | 4 | 0 |  | 3 | 4 | -1 |  | 3 | 3 | 0 |
| Arizona | 9 | 13 | -4 |  | 5 | 7 | -2 |  | 8 | 6 | 2 |  | 6 | 6 | 0 |
| Arkansas | 30 | 42 | -12 |  | 35 | 32 | 3 |  | 28 | 16 | 12 |  | 35 | 22 | 13 |
| California | 5 | 5 | 0 |  | 2 | 1 | 1 |  | 3 | 2 | 1 |  | 4 | 2 | 2 |
| Colorado | 6 | 7 | -1 |  | 3 | 3 | 0 |  | 4 | 3 | 1 |  | 4 | 3 | 1 |
| Connecticut | 11 | 13 | -2 |  | 2 | 4 | -2 |  | 7 | 4 | 3 |  | 2 | 3 | -1 |
| Delaware |  | 15 |  |  |  | 9 |  |  |  | 6 |  |  |  | 6 |  |
| District of Columbia |  | 15 |  |  |  | 2 |  |  |  | 2 |  |  |  | 1 |  |
| Florida | 10 | 19 | -9 |  | 19 | 7 | 12 |  | 10 | 8 | 2 |  | 23 | 5 | 18 |
| Georgia | 27 | 21 | 6 |  | 31 | 11 | 20 |  | 24 | 9 | 15 |  | 30 | 10 | 20 |
| Hawaii | 16 | 11 | 5 |  | 3 | 1 | 2 |  | 8 | 4 | 4 |  | 5 | 2 | 3 |
| Idaho | 11 | 12 | -1 |  | 10 | 8 | 2 |  | 10 | 7 | 3 |  | 15 | 7 | 8 |
| Illinois | 12 | 15 | -3 |  | 11 | 5 | 6 |  | 10 | 5 | 5 |  | 12 | 5 | 7 |
| Indiana | 15 | 23 | -8 |  | 16 | 16 | 0 |  | 14 | 10 | 4 |  | 19 | 11 | 8 |
| Iowa | 18 | 28 | -10 |  | 23 | 14 | 9 |  | 15 | 11 | 4 |  | 23 | 11 | 12 |
| Kansas | 18 | 25 | -7 |  | 18 | 12 | 6 |  | 13 | 9 | 4 |  | 17 | 8 | 9 |
| Kentucky | 35 | 36 | -1 |  | 66 | 23 | 43 |  | 25 | 12 | 13 |  | 45 | 13 | 32 |
| Louisiana | 24 | 39 | -15 |  | 31 | 22 | 9 |  | 23 | 13 | 10 |  | 31 | 17 | 14 |
| Maine | 8 | 10 | -2 |  | 5 | 6 | -1 |  | 4 | 3 | 1 |  | 4 | 4 | 0 |
| Maryland | 18 | 14 | 4 |  | 16 | 7 | 9 |  | 10 | 5 | 5 |  | 13 | 6 | 7 |
| Massachusetts | 1 | 8 | -7 |  | 1 | 4 | -3 |  | 1 | 2 | -1 |  | 1 | 3 | -2 |
| Michigan | 8 | 13 | -5 |  | 5 | 4 | 1 |  | 5 | 4 | 1 |  | 7 | 4 | 3 |
| Minnesota | 10 | 15 | -5 |  | 9 | 5 | 4 |  | 8 | 5 | 3 |  | 11 | 5 | 6 |
| Mississippi | 23 | 42 | -19 |  | 59 | 33 | 26 |  | 17 | 14 | 3 |  | 38 | 18 | 20 |
| Missouri | 17 | 21 | -4 |  | 27 | 12 | 15 |  | 14 | 6 | 8 |  | 33 | 10 | 23 |
| Montana | 8 | 11 | -3 |  | 5 | 4 | 1 |  | 5 | 4 | 1 |  | 5 | 3 | 2 |
| Nebraska | 23 | 39 | -16 |  | 17 | 13 | 4 |  | 16 | 12 | 4 |  | 19 | 10 | 9 |
| Nevada | 10 | 16 | -6 |  | 3 | 5 | -2 |  | 11 | 9 | 2 |  | 5 | 6 | -1 |
| New Hampshire | 7 | 9 | -2 |  | 4 | 4 | 0 |  | 2 | 5 | -3 |  | 3 | 3 | 0 |
| New Jersey |  | 19 |  |  |  | 3 |  |  |  | 7 |  |  |  | 3 |  |
| New Mexico | 7 | 5 | 2 |  | 11 | 5 | 6 |  | 5 | 2 | 3 |  | 11 | 4 | 7 |
| New York | 9 | 18 | -9 |  | 6 | 4 | 2 |  | 6 | 5 | 1 |  | 7 | 3 | 4 |
| North Carolina | 20 | 21 | -1 |  | 15 | 10 | 5 |  | 14 | 9 | 5 |  | 18 | 9 | 9 |
| North Dakota | 14 | 28 | -14 |  | 14 | 11 | 3 |  | 12 | 8 | 4 |  | 13 | 6 | 7 |
| Ohio | 17 | 21 | -4 |  | 19 | 9 | 10 |  | 13 | 6 | 7 |  | 18 | 6 | 12 |
| Oklahoma | 17 | 24 | -7 |  | 27 | 14 | 13 |  | 14 | 10 | 4 |  | 32 | 14 | 18 |
| Oregon | 8 | 7 | 1 |  | 5 | 2 | 3 |  | 5 | 3 | 2 |  | 5 | 2 | 3 |
| Pennsylvania | 12 | 12 | 0 |  | 7 | 4 | 3 |  | 7 | 4 | 3 |  | 12 | 4 | 8 |
| Rhode Island |  | 10 |  |  |  | 3 |  |  |  | 3 |  |  |  | 3 |  |
| South Carolina | 15 | 28 | -13 |  | 10 | 12 | -2 |  | 11 | 12 | -1 |  | 12 | 11 | 1 |
| South Dakota | 14 | 27 | -13 |  | 16 | 13 | 3 |  | 11 | 9 | 2 |  | 18 | 8 | 10 |
| Tennessee | 20 | 33 | -13 |  | 52 | 26 | 26 |  | 21 | 13 | 8 |  | 44 | 14 | 30 |
| Texas | 19 | 27 | -8 |  | 22 | 14 | 8 |  | 23 | 13 | 10 |  | 36 | 16 | 20 |
| Utah | 21 | 19 | 2 |  | 12 | 8 | 4 |  | 21 | 10 | 11 |  | 22 | 8 | 14 |
| Vermont | 4 | 8 | -4 |  | 4 | 4 | 0 |  | 2 | 2 | 0 |  | 3 | 3 | 0 |
| Virginia | 21 | 18 | 3 |  | 30 | 8 | 22 |  | 17 | 7 | 10 |  | 31 | 8 | 23 |
| Washington | 5 | 7 | -2 |  | 2 | 3 | -1 |  | 4 | 3 | 1 |  | 3 | 3 | 0 |
| West Virginia | 37 | 42 | -5 |  | 43 | 31 | 12 |  | 22 | 12 | 10 |  | 42 | 19 | 23 |
| Wisconsin | 10 | 15 | -5 |  | 5 | 5 | 0 |  | 7 | 5 | 2 |  | 7 | 4 | 3 |
| Wyoming | 15 | 22 | -7 |  | 12 | 17 | -5 |  | 9 | 8 | 1 |  | 12 | 13 | -1 |

Abbreviations: NP, nurse practitioner; popn, population. Data source: IQVIA Xponent®.

## FIGURE S6. Choropleth maps of rural–urban rate difference of cefdinir prescriptions by physicians and nurse practitioners by state — United States, 2022


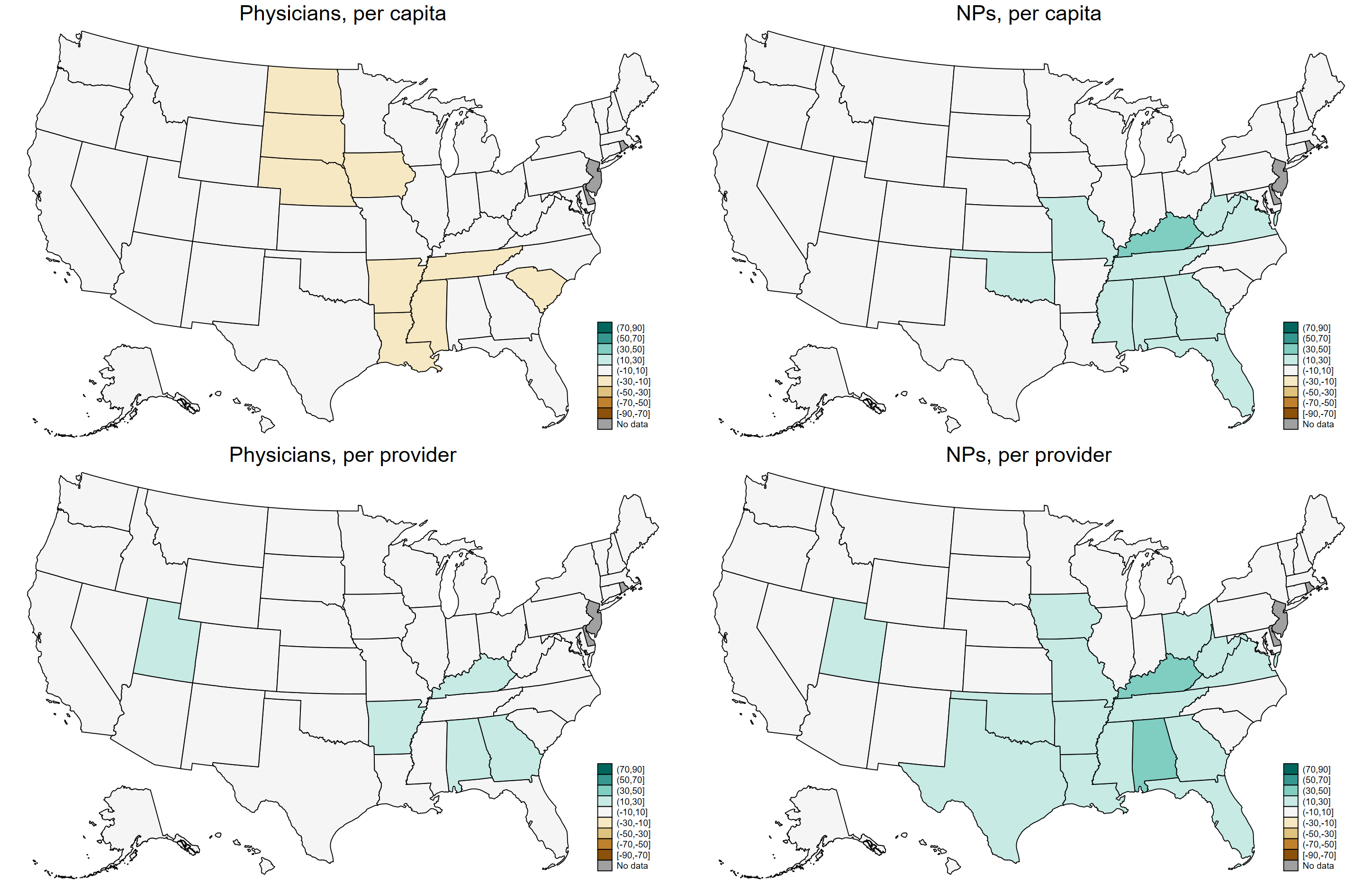


FIGURE S6. Choropleth maps of rural--urban rate difference of cefdinir by physicians and nurse practitioners (NPs) by state. Data source: IQVIA Xponent®.
